# Study of Promoter Methylation Patterns of HOXA2, HOXA5, and HOXA6 and Its Clinicopathological Characteristics in Colorectal Cancer

**DOI:** 10.3389/fonc.2019.00394

**Published:** 2019-05-21

**Authors:** Daojiang Li, Yang Bai, Zhicai Feng, Wanwan Li, Chunxing Yang, Yihang Guo, Changwei Lin, Yi Zhang, Quanyong He, Gui Hu, Xiaorong Li

**Affiliations:** ^1^Department of Gastrointestinal Surgery, The Third Xiangya Hospital of Central South University, Changsha, China; ^2^Department of Burns and Plastic Surgery of the Third Xiangya Hospital of Central South University, Changsha, China

**Keywords:** HOXA5, HOXA6, HOXA2, methylation, colorectal cancer, early stage, biomarker

## Abstract

Research on DNA methylation offers great potential for the identification of biomarkers that can be applied for accurately assessing an individual's risk for cancer. In this article, we try to find the ideal epigenetic genes involved in colorectal cancer (CRC) based on a CRC database and our CRC cohort. The top 20 genes with an extremely high frequency of hypermethylation in CRC were identified in the latest database. Remarkably, 3 HOXA genes were included in this list and ranked at the top. The percentage of methylation in the HOXA5, HOXA2, and HOXA6 genes in CRC were up to 67.62, 58.36, and 31.32%, respectively, and ranked first in CRC among all human tumor tissues. Paired colorectal tumor samples and adjacent non-tumor colorectal tissue samples and four CRC cell lines were selected for MethylTarget™ assays. The results demonstrated that CRC tissues and cells had a stronger methylation status around the 3 HOXA gene promoter regions compared with adjacent non-tumor colonic tissue samples. The Receiver operator characteristic curve (ROC) curves for HOXA genes show excellent diagnostic ability in distinguishing tissue from healthy individuals and CRC patients, especially for Stage I patients (AUC = 0.9979 in HOXA2, 0.9309 in HOXA5, and 0.8025 in HOXA6). An association analysis between the methylation pattern of HOXA genes and clinical indicators was performed and found that HOXA2 methylation was significantly associated with age, N, stage, M, lymphovascular invasion, perineural invasion, lymph node number. HOXA5 methylation was associated with age, T, M, stage, and tumor status, and HOXA6 methylation was associated with age and KRAS mutation. Notably, we found that the highest methylation of HOXA5 and HOXA2 occurs in the early stages of colorectal cancer tissues such as stage I, N0, MO, and non-invasive tissues. The methylation levels declined as tumors progressed. However, methylation level at any stage of the tumor was still significantly higher than in normal tissues (*p* < 0.0001). The mRNA of the 3 HOXA genes was downregulated in early tumor stages due to hypermethylation of CpG islands adjacent to the promoters of the genes. In addition, hypermethylation of HOXA5 and HOXA6 mainly occurred in patients < 60 years old and with MSI-L, MSS, CIMP.L and non-CIMP tumors. Together, this suggests that epigenetic silencing of 3 adjacent HOXA genes may be an important event in the progression of colorectal cancer.

## Background

Colorectal cancer (CRC) is a critical public health problem as it is the fourth most common cancer diagnosed among adults and the second leading cause of death from cancer in the developed world, and the CRC death rate is still increasing in many developing countries (1, 2). In the clinic, most CRC patients were found to be in the middle and late stages due to their indistinct symptoms. Advanced patients with distant metastases (T, N, and M1 stages) have an average 5-year survival rate of ~5%, while those with early stage CRC in which the tumor has not breached the muscularis mucosa (TNM stage Tis, N0, M0) have a 5-year survival rate of 100%; even in patients with invasive cancer of T1 or T2 stages, the survival rates can reach ~90% (3–5). Therefore, early diagnosis and treatment can greatly improve the prognosis of this aggressive neoplasm.

In our previous study (6), based on TCGA (The Cancer Genome Atlas) data, we performed a comprehensive bioinformatics analysis of mRNA expression in colorectal cancer. We found that the genes located on chromosome 20q were frequently upregulated in colorectal cancer, and these genes can be studied as optimal candidates for oncogenes. However, tumorigenesis is not only a result of oncogene activation but also tumor suppressor inactivation. We now understand that tumor suppressor gene silencing by promoter hypermethylation is an important mechanism in carcinogenesis (7, 8). There are numerous examples of this mechanism in colorectal cancer, such as the mismatch repair gene human mutL homolog 1 (MLH1) with notable methylation and aberrant patterns of DNA methylation leading to activation of the Wnt pathway by silencing the secreted frizzled-related protein (SFRP) family of genes (9, 10). In addition, DNA methylation is more frequent and easier to detect than some genetic changes, which indicate that it may be more suitable for clinical applications (9). In this article, based on the latest colorectal cancer database and our experiments, we analyze the methylation status in three HOXA genes including HOXA2, HOXA5, and HOXA6 and its clinical application.

## Methods

### Cell Lines and Tissue Samples

The colorectal cell lines SW620, HCT-116, HT-29, and SW480 have been described previously (11). Human CRC cell lines (SW620, SW480) were cultured with L15 medium (KeyGEN BioTECH, Nanjing, China), supplemented with 10% fetal bovine serum (FBS) (Biological Industries, Israel), and HCT116, HT-29 were cultured in McCoy‘s5A medium (KeyGEN BioTECH, Nanjing, China) with 10% FBS. Cells were grown in a 5% CO2 cell culture incubator at 37°C. Clinical samples of human colorectal cancer tissues and paired-adjacent non-tumor tissues that were farther than 5 cm from the tumors were obtained from patients who underwent radical resection at the Third XiangYa Hospital of Central South University (Hunan, China) under informed consent and approval by the Ethics Committee of Central South University. Fresh samples were snap frozen in liquid nitrogen immediately after resection and stored at −80°C. More details about clinical material including size, sex, age, lymph node, TMN, pathology, differentiation, MSI, and organ can be found in Supplemental Table 1.

### RNA Purification and Reverse Transcription-PCR

Total RNA was extracted using the TRIZOL reagent (Invitrogen, USA). The reverse transcription reactions were performed using ReverTra Ace qPCR RT Master Mix with gDNA Remover (TOYOBO). cDNA was amplified by KOD SYBR® qPCR Mix (TOYOBO) on a LightCycler® 480II System (Roche) according to the manufacturer's instructions. The primer sequences for target genes used in the reactions are shown in Table 1.

**Table 1:** The primer sequences in this article.

**PrimerName**	**Primer**
**Table 1–1 : Primers used for the MethylTarget**™**assays**	
HOXA2_F	GGGTATTYGGGYGGTTGTAgg
HOXA2_R	AATACCTAACATCTTTTCCCCCTATC
HOXA5_1F	AATATAGTTGAGAGGTAAGTGGAGTTTTT
HOXA5_1R	TAAAACATTACAACCCRAAATAAACTCC
HOXA5_2F	TATTYGTTGGAGGTAGGGTTTATYGT
HOXA5_2R	CCCCCAATCCTCTACATCCT
HOXA5_3F	AGAGGAGGAAGGAGGAAGGT
HOXA5_3R	CCCCTACCAACAACRAACAAAC
HOXA5_4F	ACCCCCAAAATTCAAAATCCTAC
HOXA5_4R	GGAAGGGGGAGGAAGTAAAA
HOXA6_1F	TTGTAGGATTGTGATTTGTTGTGT
HOXA6_1R	aaCRaacTAAAAACTATTTATAACTTTACTACCTC
HOXA6_2F	AAGTGTAGGTAGTTTTYGGGGTTTTT
HOXA6_2R	ACCAACTACCCCTCTACCAAAC
**Table 1–2: Primers used for the qRT-PCR assays**	
HOXA5-F	AGATCTACCCCTGGATGCGC
HOXA5-R	CCTTCTCCAGCTCCAGGGTC
HOXA2-F	CCCCTGTCGCTGATACATTTC
HOXA2-R	TGGTCTGCTCAAAAGGAGGAG
HOXA6-F	ACTACCTGCACTTTTCTCCCG
HOXA6-R	GCTCGTGTACTTCCGGTCG
**Table 1–3: Bisulfite sequencing (or restriction) PCR, Product size: 300, CpGs in product: 27**	
HOXA5(BSP)-F	TTATAATGGGTTGTAATTTTAATT
HOXA5(BSP)-R	AACATATACTTAATTCCCTCCTAC

### Treatment With 5-Aza-2′-Deoxycytidine (5-Aza)

Cells were grown in appropriate culture conditions. For demethylation treatment, colorectal cell lines were treated with 5-Aza-2′-deoxycytidine (Sigma, USA) for 96 h (5, 10, and 15 μmol/L) with daily replacements of the drug and medium. Cells without drug treatment were used as the control group.

### DNA Extraction and Bisulfate Treatment

DNA was isolated with the Easypure Genomic DNA kit (TransGen Biotech, Beijing, China) according to the manufacturer's instructions. The concentration of DNA was assessed by spectrophotometry and confirmed by gel electrophoresis before storage of DNA at −20°C. The EZ DNA Methylation Gold™ Kit (Zymo Research Corporation, CA, USA) was used to convert all unmethylated cytosine to uracil.

### Targeted Bisulfite Sequencing Assay

MethylTarget™ assays (targeted bisulfite sequencing) developed by Genesky BioTech (Shanghai, China) were carried out as previously described (12, 13). CpG islands adjacent to promoter regions of 3 HOXA genes were assessed by Genesky BioTech (Shanghai, China). Briefly, in this work the putative human core promoters were identified as regions within a window of 2,000/+1,000 bp relative to the predicted TSS locations. The parameters for methylated CpG island assessment are: Observed/Expected ratio >0.60, Percent C + Percent G > 50.00%, Length >200 bp. Fragment number evaluation: Each CpG island is expected to design a pair of primers for detection, for longer CpG islands (200 bp-1K, 1 pair, 1K-2K, 2 pairs, and so on), and at least 10 CpG sites were detected in each fragment. One CpG region from the CpG islands of HOXA2A, 4 regions from HOXA5 and 2 regions from HOXA6 were sequenced (the details including the relative distance to the transcriptional start site, primer, size, etc., for these CpG regions can be found in Tables 1, 2). Genomic DNA was converted with bisulfite treatment, and PCR reactions were performed to amplify the targeted DNA sequences. The products were sequenced on an Illumina MiSeq Benchtop Sequencer (CA, USA).

**Table 2:** Details of the CpG regions in the CpG islands of HOXA2, HOXA5, and HOXA6.

**Target**	**TSS**	**Start**	**End**	**Length**	**Target Strand**	**Distance 2TSS**
HOXA2_	27142394	27141833	27142061	229	+	333
HOXA5_1	27183287	27184397	27184606	210	+	−1,319
HOXA5_2	27183287	27183537	27183722	186	+	−435
HOXA5_3	27183287	27182501	27182769	269	+	518
HOXA5_4	27183287	27182432	27182185	248	-	855
HOXA6_1	27187393	27187381	27187595	215	+	−202
HOXA6_2	27187393	27187055	27187301	247	+	92

*Start, The starting position of the product on the reference genome; TSS, The mRNA transcription initiation site; End, The end position of the product on the reference genome; Length, the product length; Target strand, The product orientation; Distance2TSS, The distance from the product to the TSS*.

### Bisulfite Sequencing PCR (BSP)

For Bisulfite-polymerase chain reaction, BSP primer was designed by MethPrimer 2.0, then the bisulfate modified DNA was amplified with forward and reverse primers for target genes (bisulfite sequencing or restriction PCR, BSP) (Table 1). Amplified PCR products were purified and cloned into pMD19-T (TaKaRa, Dalian, China). For each gene, five clones of each sample were sequenced. Percentage of methylation was calculated by software Biq-analyzer.

### Bioinformatics

COSMIC (14), a database that catalogs somatic mutations in human cancer, was used to assess the methylation status in human cancer tissues. To assess the expression levels of HOXA5, HOXA2, and HOXA6, the following datasets were used: GEO GSE4183 and GSE8671 and TCGA for colorectal cancer (15). Data concerning the methylation status, patient information and samples were downloaded from the TCGA database (https://cancergenome.nih.gov/). MethPrimer (http://www.urogene.org/methprimer/index.html) was used for predicting methylation islands.

### Statistics

The GraphPad Prism 7 software was used to analyze the data. Statistical significance was tested by a Student's *t*-test or a chi-square test as appropriate. If multiple hypotheses are tested, the Bonferroni correction was used to adjust the significance level for each individual test to a stricter one; One-way ANOVA was used to compare three or more unmatched groups; error bars in the figures represent SD or SEM. Box-whisker plots depict the mean, 1st and 3rd quartiles, minimum, and maximum. Scatter plots and scatter plots with bars show the mean or mean + SD. Spearman's correlation coefficient (*r*) was used to determine the correlation. Receiver operator characteristic curves (ROCs) were constructed based on the level of HOXA gene methylation. The data were considered not significant if *p* > 0.05.

## Results

### Extremely High Frequency of Hypermethylation of HOXA2, HOXA5, and HOXA6 Genes in CRC Tissue

**Figure 1:**
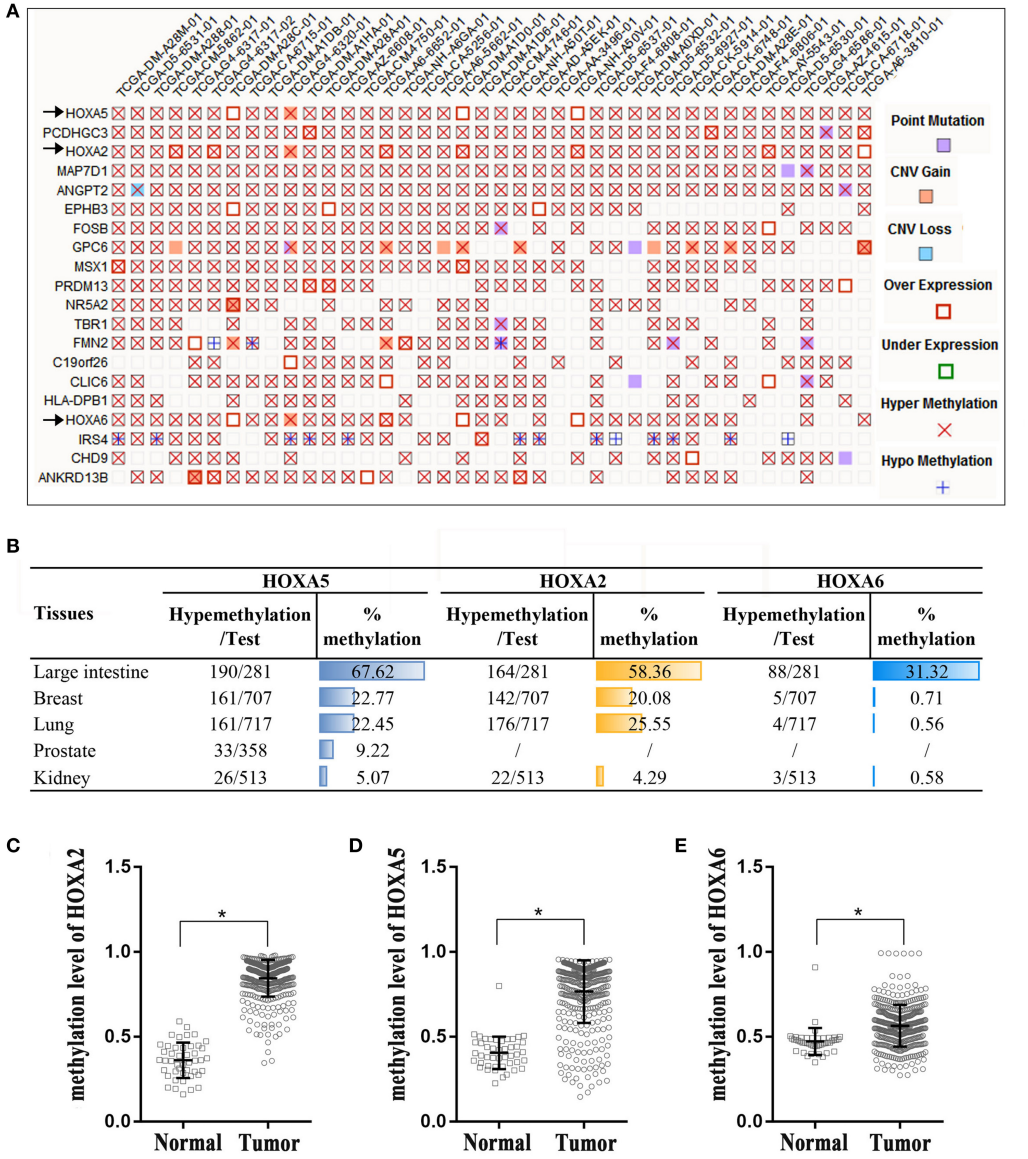
High percentage of hypermethylation of the HOXA2, HOXA5, and HOXA6 genes in colorectal cancer tissue. **(A)** A “Mutation Matrix” plot between genes and samples for colorectal tissue that contains the top 20 ranked genes (rows) and TCGA samples (columns) with each box representing a Gene-Sample combination. HOXA2, HOXA5, and HOXA6 ranked 1, 3, and 17, respectively (only some TCGA samples are listed here; more details can be found on the COSMIC website). **(B)** The hypermethylation percentages (% me) are represented as histograms across the different primary tissue types, and the percentage of hypermethylation of HOXA2, HOXA5, and HOXA6 in the large intestine rank first. Test: Total number of samples analyzed (only some cancer samples are listed here; more details can be found on the COSMIC website). **(C–E)** HOXA2, HOXA5, and HOXA6 genes show increased methylation levels in colorectal cancer tissues compared with normal tissues (Unpaired *t*-test, ^*^all *p* < 0.0001).

The Catalog of Somatic Mutations in Cancer (COSMIC) (14), the world's largest and most comprehensive database of somatic mutations in human cancer, was used to analyze DNA methylation variations in colorectal adenocarcinoma. Among the top 20 genes with the highest degree of methylation in colorectal cancer, we found that there are three HOXA genes. HOXA5, HOXA2, and HOXA6 rank first, third and seventeenth, respectively (Figure 1A). We next expanded our methylation analysis to different human cancer tissue types. We found that the methylation percentages of HOXA5, HOXA2, and HOXA6 in CRC tissue were up to 67.62, 58.36, and 31.32%, respectively, and ranked first in CRC among all cancer types analyzed (Figure 1B). The DNA methylation profiles of the 3 HOXA gene promoter regions were downloaded from TCGA, and the results demonstrated that the methylation levels of HOXA5, HOXA2, and HOXA6 were significantly elevated in cancer tissues compared with normal tissues (*p* < 0.0001) (Figures 1C–E). This indicated that hypermethylation of HOXA5, HOXA2, and HOXA6 is an important event in colorectal cancer.

### Targeted Bisulfite Sequencing to Assess the Methylation Features of HOXA5, HOXA2, and HOXA6 in Colorectal Cancer Samples and Cells

**Figure 2:**
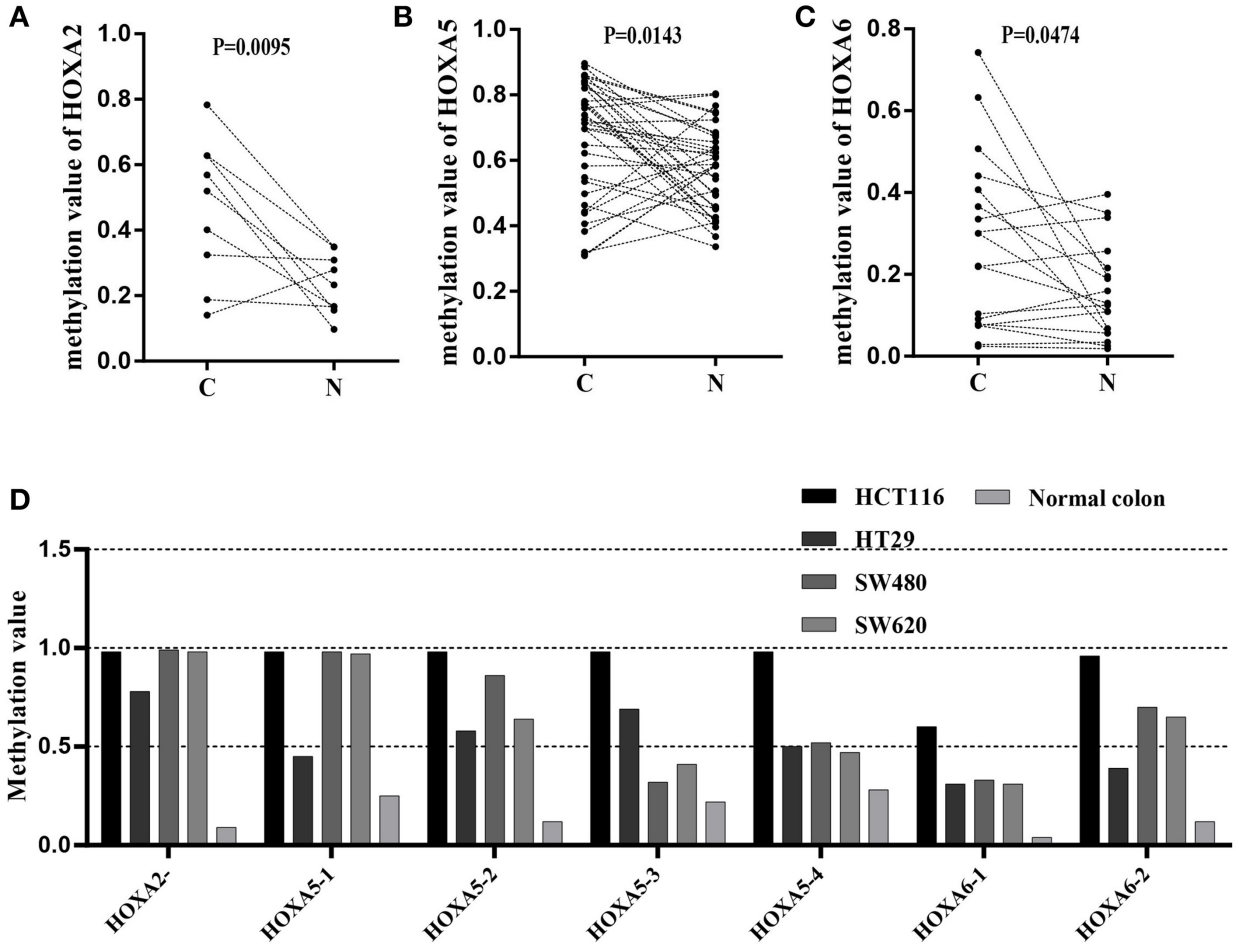
Results of MethylTarget TM assays in nine paired non-cancerous tissues and CRC tissues and CRC cells. **(A–C)** One CpG region from the CpG islands of HOXA2, 4 from HOXA5, and 2 from HOXA6 were sequenced in 9 paired non-cancerous tissues and CRC tissues. **(D)** One CpG region from the CpG islands of HOXA2, 4 from HOXA5, and 2 from HOXA6 were sequenced in four colorectal cancer cells and normal colon tissue.

To further confirm that the promoters of HOXA5, HOXA2, and HOXA6 have aberrant methylation patterns in CRC, nine paired colorectal adenocarcinoma samples and adjacent non-tumor colorectal tissue samples and four CRC cell lines were selected for MethylTarget™ assays. According to the CpG islands adjacent to the promoter regions of the three genes, 1 CpG region from HOXA2 (HOXA2-), 4 regions from HOXA5 (HOXA5-1, HOXA5-2, HOXA5-3, and HOXA5-4), and 2 regions from HOXA6 (HOXA6-1 and HOXA6-2) were sequenced (Table 2). The results demonstrated that, compared with adjacent non-tumor colonic tissue samples, CRC tissues exhibited stronger methylation patterns (Figures 2A–C), an identical phenomenon was observed in CRC cells (Figure 2D). To better characterize the methylation of the HOXA genes, we also assessed the methylation level of each CpG site. We found that almost every CpG site (18/19) in HOXA2 in CRC tissue had a significantly stronger methylation pattern than corresponding non-cancerous tissues (Table 3). This was consistent with the results from the TCGA CRC datasets, which showed that HOXA2 promoter methylation was increased most significantly in CRC compared to normal tissues (Figure 1C). Howerver, no such perfect results are presented on the HOXA5 and HOXA6 genes, although in general the HOXA5 and HOXA6 genes are significantly methylated in tumor tissues (Supplemental Table 2), which may be due to the larger CpG island region of HOXA5 and HOXA6 and also indicates that HOXA2 gene hypermethylation is more obvious in colorectal cancer.

**Table 3:** Methylation level of each CG site in the CpG island regions of HOXA2 gene in 9 paired colorectal adenocarcinoma samples and adjacent non-tumor colonic tissue samples.

**Target**	**Position**	**Type**	***P-*value(T-test)**	**OR(L95-U95)(Logistic)**	**MethylDiff**	***C* mean**	***N* mean**
HOXA2_	23	CG	0.0184384	1.0836(1.0020–1.1718)	0.233133	0.497291	0.264158
HOXA2_	26	CG	0.0131794	1.0982(1.0047–1.2004)	0.246741	0.442534	0.195793
HOXA2_	29	CG	0.0175961	1.0853(1.0036–1.1738)	0.239593	0.4711	0.231507
HOXA2_	36	CG	0.0097988	1.1067(1.0055–1.2181)	0.260802	0.441725	0.180923
HOXA2_	44	CG	0.0116775	1.1049(1.0060–1.2136)	0.239451	0.401936	0.162485
HOXA2_	46	CG	0.0138777	1.0922(1.0043–1.1879)	0.243499	0.448467	0.204969
HOXA2_	54	CG	0.0132013	1.0891(1.0062–1.1789)	0.24928	0.437937	0.188657
HOXA2_	56	CG	0.0099816	1.1039(1.0047–1.2129)	0.269025	0.424603	0.155577
HOXA2_	63	CG	0.0164194	1.0872(1.0032–1.1782)	0.238949	0.449903	0.210954
HOXA2_	73	CG	0.0117399	1.0918(1.0033–1.1882)	0.228761	0.506655	0.277894
HOXA2_	77	CG	0.0089776	1.0912(1.0052–1.1845)	0.242318	0.520224	0.277905
HOXA2_	83	CG	0.0134568	1.0944(1.0045–1.1923)	0.244307	0.471639	0.227332
HOXA2_	86	CG	0.0127031	1.0962(1.0046–1.1962)	0.241186	0.458494	0.217308
HOXA2_	91	CG	0.0107993	1.0890(1.0052–1.1798)	0.229304	0.503858	0.274554
HOXA2_	104	CG	0.0179164	1.0826(1.0026–1.1690)	0.204942	0.481185	0.276243
HOXA2_	153	CG	0.024104	1.0787(1.0004–1.1632)	0.190415	0.45092	0.260505
HOXA2_	165	CG	0.0092004	1.0988(1.0049–1.2016)	0.225584	0.517545	0.291961
HOXA2_	168	CG	0.051281	1.0790(0.9932–1.1723)	0.136829	0.368444	0.231616
HOXA2_	203	CG	0.015797	1.0829(1.0024–1.1698)	0.219589	0.530772	0.311183

### The Association Between the Methylation Patterns of HOXA5, HOXA2, and HOXA6 and Clinical Indicators for CRC Patients

**Figure 3:**
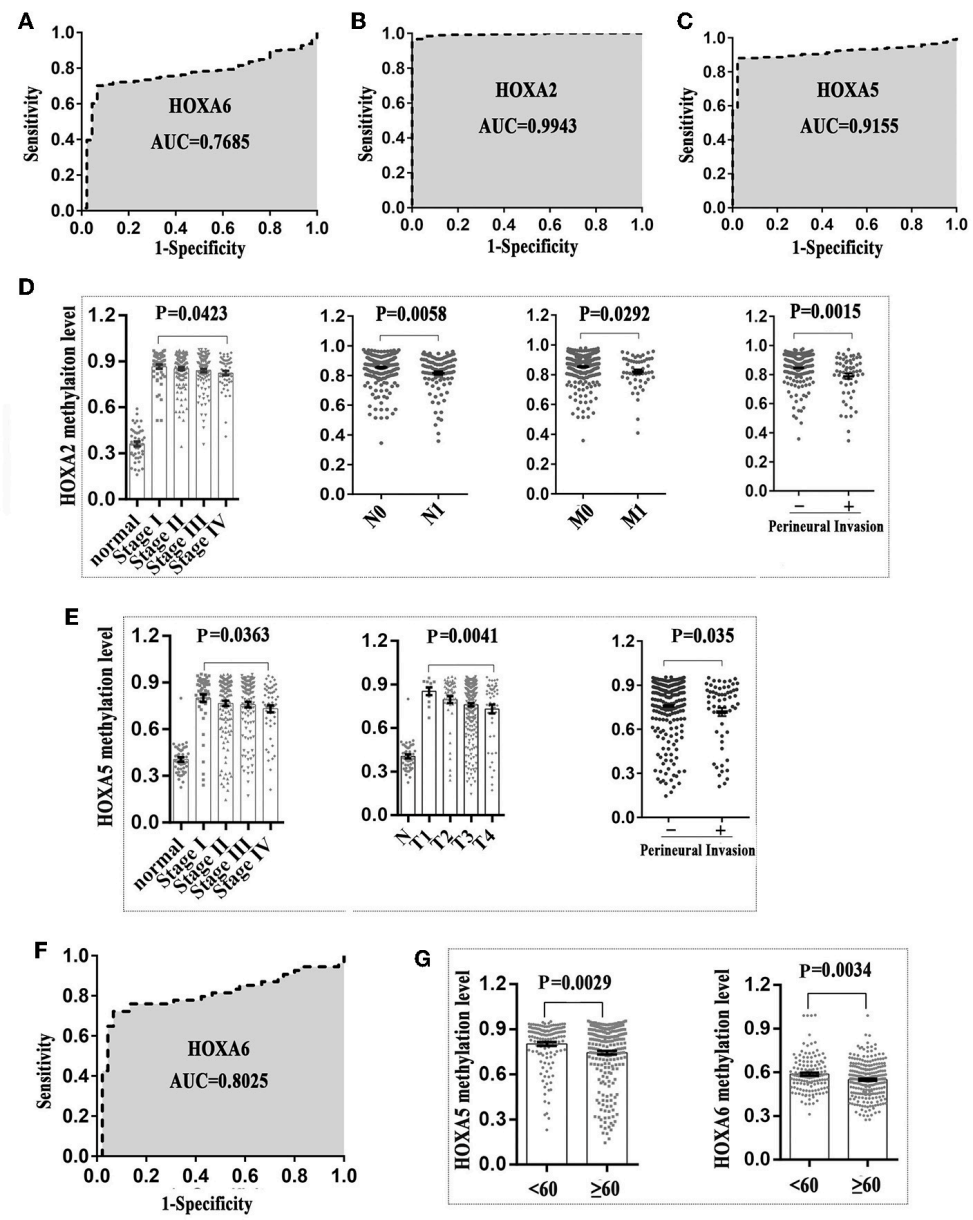
The methylation patterns of 3 HOXA genes can serve as independent clinical biomarkers for colorectal patients. **(A–C)** Receiver operator characteristic curves were used to assess the clinical diagnostic utility of DNA methylation of HOXA6, HOXA2, and HOXA5 for the prediction of CRC. **(D)** HOXA2 showed the highest methylation level in early stages of colorectal cancer including stage I, N0, MO, and no perineural invasion. The methylation level declined with tumor progression (Unpaired *t*-test, compared with normal tissue, *p* < 0.0001 in all stages). **(E)** HOXA5 showed the highest methylation level in early stages of colorectal cancer including stage I, T1, and no perineural invasion. The methylation level declined with tumor progression. **(F)** The receiver operator characteristic curves were used to assess the clinical diagnostic utility of DNA methylation of HOXA6 in distinguishing tissues from healthy individuals and Stage I patients. **(G)** HOXA5 and HOXA6 genes showed increased methylation levels in patients younger than 60.

First, we generated a receiver ROC to assess the clinical utility of DNA methylation for the prediction of CRC. The methylation level of HOXA genes was highly discriminative between normal tissues and CRC tissues (area under the curve = 0.9155 in HOXA5, 0.9943 in HOXA2, and 0.7685 in HOXA6) (Figures 3A–C). This suggests that methylation of HOXA genes, especially HOXA2 and HOXA5, may be a useful marker to screen individuals with a high risk of CRC. Next, we analyzed the relationship between the methylation levels and clinicopathological features. First, all CRC tissue samples were divided into two groups according to the mean value of methylation at the HOXA genes. Then, 20 clinical indicators, including age, weight, vascular, and lymphovascular invasion, stage, M, T, etc., were analyzed with the chi-square test. We found that HOXA2 methylation was significantly associated with age (*high methylation rate in patients*<*60 is 53.0%*, ≥*60 is 66.7%, P* = *0.009*), *N* [*the Bonferroni correction test each individual hypothesis at a significance level of 0.05/3* = *0.0167, high methylation rate in N0 is 68.4%, N1 is 47.6% and N2 is 64.3%; N0 vs. N1 (P* = *0.0004*<*0.0167), N0 vs. N2 (P* = *0.5248, no statistical significance), N1 vs. N2 (P* = *0.0303, no statistical significance)*], M (*high methylation rate in M0 is 66.3%, M1 is 45.3%, p* = *0.005*), lymphovascular invasion (*high methylation rate in yes is 51.4%, no is 64.9%, p* = *0.018*), perineural invasion (*high methylation rate in yes is 42.4%, no is 63.5%, p* = *0.005*), lymph node number [*the Bonferroni correction test each individual hypothesis at a significance level of 0.05/3* = *0.0167, high methylation rate in 0 is 67.2%, 1–3 is 47.4% and* ≥*4 is 62.7%; 0 vs. 1–3 (P* = *0.0012*<*0.0167), 0 vs*. ≥*4 (P* = *0.5030, no statistical significance), 1–3 vs*. ≥*4 (P* = *0.0542, no statistical significance)*], and Stage [*the Bonferroni correction test each individual hypothesis at a significance level of 0.05/6* = *0.0083, high methylation rate in I is 72.2%, I is 67.1%, III is 58.5%, and IV is 46.30%; I vs. IV (P* = *0.006*<*0.0083),II vs.IV (P* = *0.0074*<*0.0083), others are not statistically significant)*] (Table 4). HOXA5 methylation was associated with age (*high methylation rate in patients*<*60 is 72.1%*, ≥*60 is 58.7%, P* = *0.011*), T [*the Bonferroni correction test each individual hypothesis at a significance level of 0.05/6* = *0.0083, high methylation rate in T1 is 81.8%, T2 is 79.6%, T3 is 60.0%, and T4 is 58.8%; T2 vs. T3 (P* = *0.0081*<*0.0083), others are not statistically significant)*], M (*high methylation rate in M0 is 65.9%, M1 is 47.2%, p* = *0.013*), stage [*the Bonferroni correction test each individual hypothesis at a significance level of 0.05/6* = *0.0083, high methylation rate in I is 79.6%, II is 63.6%, III is 61.0%, and IV is 50.0%; I vs. IV (P* = *0.0023*<*0.0083), others are not statistically significant)*], and tumor status (*high methylation rate in tumor free is 67.1%, with tumor is 54.9%, P* = *0.046*) (Supplemental Table 3). HOXA6 methylation was associated with age (*high methylation rate in patients*<*60 is 55.1%*, ≥*60 is 44.0 %, P* = *0.043*), KRAS mutation (*high methylation rate in yes is 25.0%, no is 67.9%, P* = *0.001*) (Supplemental Table 4). Notably, we found that the highest methylation of HOXA5 and HOXA2 occurs in early stage colorectal cancer tissues such as stage *I and II*, N0, MO, and non-invasive. The methylation levels declined as the tumor progressed. To understands our conclusions more intuitively and strengthen our conclusions, we performed unpaired *t*-tests on the pathological data and obtained similar results as shown in Figures 3D,E. Although the degree of methylation decreased with tumor progression, it was still significantly higher than in normal tissues (*p* < 0.0001). Together, these findings suggested that methylation events occurring may be an early event in colorectal cancer development. Further, the HOXA genes' ROC curves showed excellent diagnostic ability to distinguish tissue from healthy individuals and Stage I patients (AUC = 0.9979 in HOXA2, 0.9309 in HOXA2 and 0.8025 in HOXA6) (Figure 3F and Supplemental Figures 1A,B). In addition, we also found that HOXA5 and HOXA6 were more likely to be methylated in patients < 60 years of age (Figure 3G and Supplemental Tables 3, 4).

**Table 4:** The relationship between the methylation levels of HOXA2 and clinicopathological factors.

**Factor#**	**No**.	**HOXA2**		***P***
		**Low, *n* (%)**	**High, *n* (%)**	
**AGE**				
< 60	136	64 (47.06)	72 (52.94)	0.009^*^
≥60	252	84 (33.33)	168 (66.67)	
**GENDER**				
Male	210	75 (35.71)	133 (64.29)	0.296
Female	178	73 (41.01)	105 (58.99)	
**HEIGHT**				
< 170	138	56 (40.58)	82 (59.42)	0.811
≥170	151	59 (39.07)	92 (60.93)	
**WEIGHT**				
< 80	156	60 (38.46)	96 (61.54)	0.816
≥80	152	61 (40.13)	91 (59.87)	
**RACE**				
Asian	12	3 (25.00)	9 (75.00)	0.174
Black	62	19(30.65)	43 (69.35)	
White	280	116(41.43)	164(58.57)	
**T**				
T1	11	3 (27.27)	8 (72.73)	0.187
T2	54	15 (27.78)	39 (72.22)	
T3	270	105 (38.89)	165 (61.11)	
T4	51	24 (47.06)	27 (52.94)	
**N**				
N0	212	67 (31.60)	145 (68.40)	0.002^*^^a^
N1	103	54 (52.43)	49 (47.57)	
N2	70	25 (35.71)	45 (64.29)	
**M**				
M0	264	89 (33.71)	175 (66.29)	0.005^*^
M1	53	29 (54.72)	24 (45.28)	
**STAGE**				
I	54	15 (27.78)	39 (72.22)	0.016^*^^a^
II	143	47 (32.87)	96 (67.13)	
III	118	49 (41.53)	69(58.47)	
IV	54	29 (53.70)	25 (46.30)	
**LYMPHOVASCULAR INVASION**				
Yes	107	52 (48.60)	55 (51.40)	0.018^*^
No	231	81 (35.06)	150 (64.94)	
**VASCULAR INVASION**				
Yes	78	33 (42.31)	45 (57.69)	0.508
No	254	96 (37.80)	158 (62.20)	
**PERINEURAL INVASION**				
Yes	59	34 (57.63)	25 (42.37)	0.005^*^
No	170	62 (36.47)	108 (63.53)	
**KRAS Mutation**				
Yes	28	6 (21.43)	22 (78.57)	0.152
No	28	12 (42.86)	16 (57.14)	
**LYMPH NODES NO.HE**				
0	192	63 (32.81)	129 (67.19)	0.005^*^^a^
1–3	95	50 (52.63)	45 (47.37)	
≥4	67	25(37.31)	42 (62.69)	
**LYMPH NODES NO**				
< 18	168	60 (35.71)	108 (64.29)	0.327
≥18	189	78 (41.27)	111 (58.73)	
**TUMOR STATUS**				
Tumor free	246	97 (39.43)	149 (60.57)	0.794
With tumor	82	31 (37.80)	51 (62.20)	
**TUMOR SIZE**				
< 0.5	123	42 (34.15)	81 (65.85)	0.059
≥0.5	108	50 (46.30)	58 (53.70)	
**SURGICAL MARGIN**				
R0	256	100 (39.06)	156 (60.94)	0.423
R1	5	1 (20.00)	4 (80.00)	
R2	7	4 (57.14)	3 (42.86)	
**HISTORY**				
Yes	31	10 (32.26)	21 (67.74)	0.566
No	357	138 (38.66)	219 (61.34)	
**TUMOR SITE**				
Ascending colon	55	17 (30.91)	38 (69.09)	0.003^*^^a^
Cecum	74	22 (29.73)	52 (70.27)	
Descending colon	14	6 (42.86)	8 (57.14)	
Rectosigmoid junction	46	26 (56.52)	20 (43.48)	
Transverse colon	47	15(31.91)	32(68.09)	
Rectum	45	10 (22.22)	35 (77.78)	
Sigmoid colon	88	43 (48.86)	45 (51.14)	

* Means the result has statistical significance, # more details about explanation for Factor can be found in the TCGA database.

a *If multiple hypotheses are tested, the Bonferroni correction was used to adjust the significance level for each individual test to a stricter one, the Bonferroni correction test each individual hypothesis at a significance level of 0.05/m. m is the number of hypotheses*.

### The Association Between the Methylation Patterns of HOXA5, HOXA2, and HOXA6 and CIMP or MSI Status

**Figure 4:**
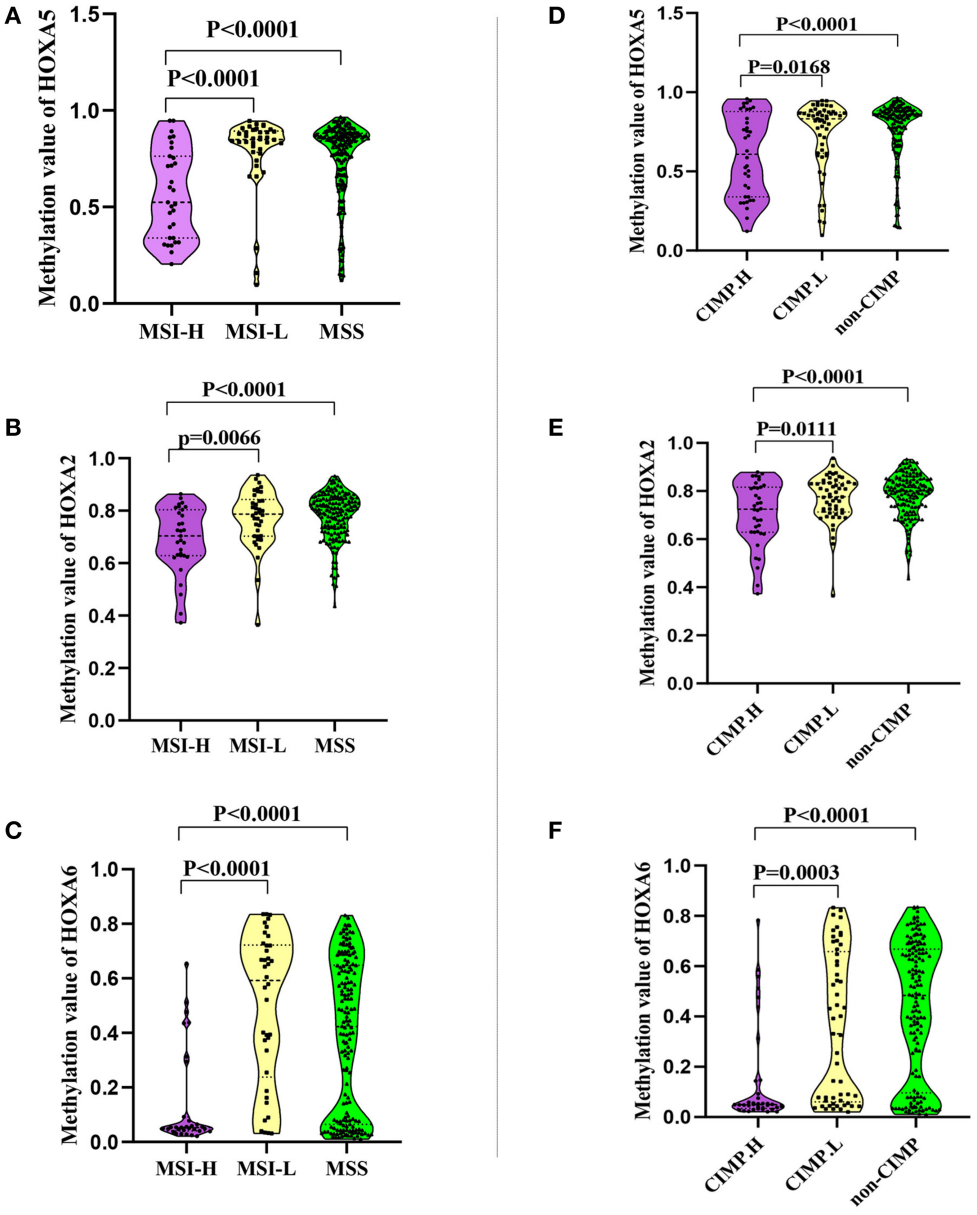
The association between the methylation patterns of 3 HOXA genes and microsatellite instability or CpG island methylator phenotype status. **(A–C)** Normalized methylation value of HOXA5, HOXA2, and HOXA6 in colorectal tumor with MSI-H, MSI-L, and MSS status. The result presented as violin plot showing all points. **(D–F)** Normalized methylation value of HOXA5, HOXA2, and HOXA6 in colorectal tumor with CIMP.H, CIMP.L, and not-CIMP status. The result presented as violin plot showing all points.

In this study we also evaluated the association between the methylation patterns of 3 HOXA genes and microsatellite instability (MSI) or CpG island methylator phenotype (CIMP) status. The TCGA colorectal tissues (15) including MSI status, CIMP status, and methylation value was downloaded. Firstly, 235 colorectal samples consist of 31 with high levels of MSI (MSI-H), 38 with low levels of MSI (MSI-L), and 166 with microsatellite-stable (MSS) were selected for MSI analysis (Supplemental Table 5). Analysis of the promoter DNA methylation profiles of HOXA5 by One-way ANOVA, we found a clear distinction in three MSI groups (*F* = 11.66, *P* < 0.0001). A similar pattern was observed for HOXA2 (*F* = 11.48, *P* < 0.0001) and HOXA6 methylation value (*F* = 16.16, *P* < 0.0001). Furthermore, through unpaired *t*-test, we identified a statistically significant upregulation of HOXA5 methylation value from MSI-H to MSI-L (*P* < 0.0001) or MSS (< 0.0001), no statistically significant differences between MSI-L and MSS (*P* = 0.2785) (Figure 4A). An identical pattern was observed in HOXA2 (MSI-H vs. MSI-L, *P* = 0.0066; MSI-H vs. MSS, P < 0.0001) and HOXA6 (MSI-H vs. MSI-L, *P* < 0.0001; MSI-H vs. MSS, *P* < 0.0001) (Figures 4B,C). All these results suggest that the hypermethylation of 3 HOXA gens mainly occurred in MSI-L and MSS samples, especially MSS patients. Then, 236 colorectal tumors were classified as three subgroups including CIMP high (CIMP.H, *n* = 36), CIMP low (CIMP.L, *n* = 53), and non-CIMP (*n* = 147), as previously studies described (15, 16) (Supplemental Table 6). Interesting, in line with the result of MSI analysis, the promoter DNA methylation profiles of HOXA5 (*F* = 8.644, *P* = 0.0002), HOXA2 (*F* = 8.639, *P* = 0.0002), and HOXA6 (*F* = 16.66, *P* < 0.0001) were markedly different in 3 CIMP group (One-way ANOVA). Furthermore, analogous to the MSI result, we found that CIMP.L and non-CIMP group, especially non-CIMP, have a higher methylation profile for HOXA5 (CIMP.H vs. CIMP.L, *P* = 0.0168; CIMP.H vs. non-CIMP, *P* < 0.0001), HOXA2 (CIMP.H vs. CIMP.L, *P* = 0.0111; CIMP.H vs. non-CIMP, *P* < 0.0001), and HOXA6 (CIMP.H vs. CIMP.L, *P* = 0.0003; CIMP.H vs. non-CIMP, *P* < 0.0001) (Figures 4D–F). No clear distinctions between CIMP.L and non-CIMP samples (HOXA5, *P* = 0.2574; HOXA2, *P* = 0.3435; HOXA6, *P* = 0.1017). Together, these findings indicating promoter DNA hypermethylation of HOXA5, HOXA2, and HOXA6 is associated with MSI-L, MSS, CIMP.L and non-CIMP tumors, especially in MSS and non-CIMP samples.

### Promoter Methylation Is Responsible for the Silencing of 3 HOXA Genes in Colorectal Cancer

**Figure 5:**
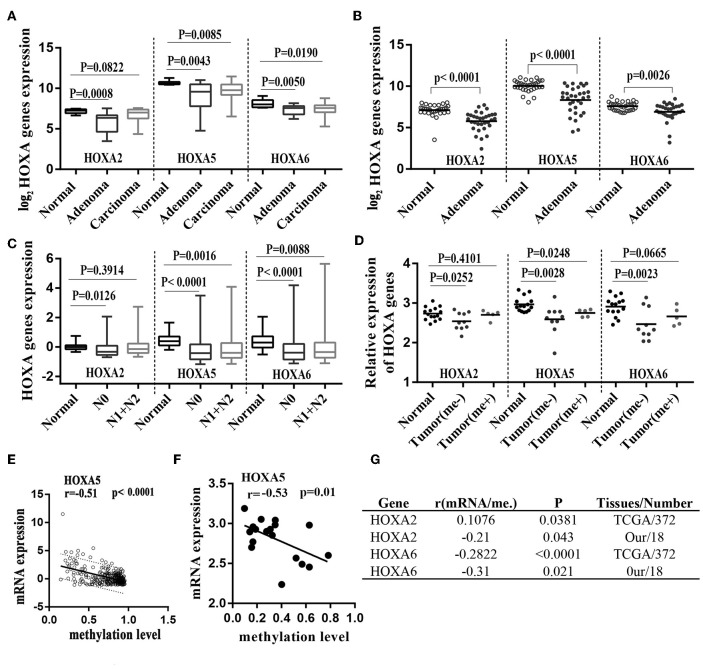
Hypermethylation of HOXA2, HOXA5, and HOXA6 correlates with decreased expression of theses gene during colorectal cancer. **(A)** Normalized mRNA expression of 3 HOXA genes in adenoma, carcinoma and normal tissue presented as box-whisker plots (GEO: GSE4183). **(B)** Normalized mRNA expression of 3 HOXA genes in adenoma and normal tissue presented as a scatter plot (bar: mean) (GEO: GSE8671). **(C)** Normalized mRNA expression of 3 HOXA genes in CRC tumors with or without lymph node metastasis and normal tissue presented as box-whisker plots (TCGA-CRC). **(D)** Relative mRNA expression of 3 HOXA genes in 15 paired colorectal cancer tissues (15 tumor tissues including 5 patients with metastasis and 10 without) and adjacent normal tissues presented as a scatter plot (bar: mean). **(E–G)** The expression of the HOXA5 and HOXA6 genes is significantly inversely correlated with their methylation values in TCGA tissues and our own tissues (analyzed with Pearson correlation analysis, and the 95% prediction band is shown in **A**).

**Figure 6:**
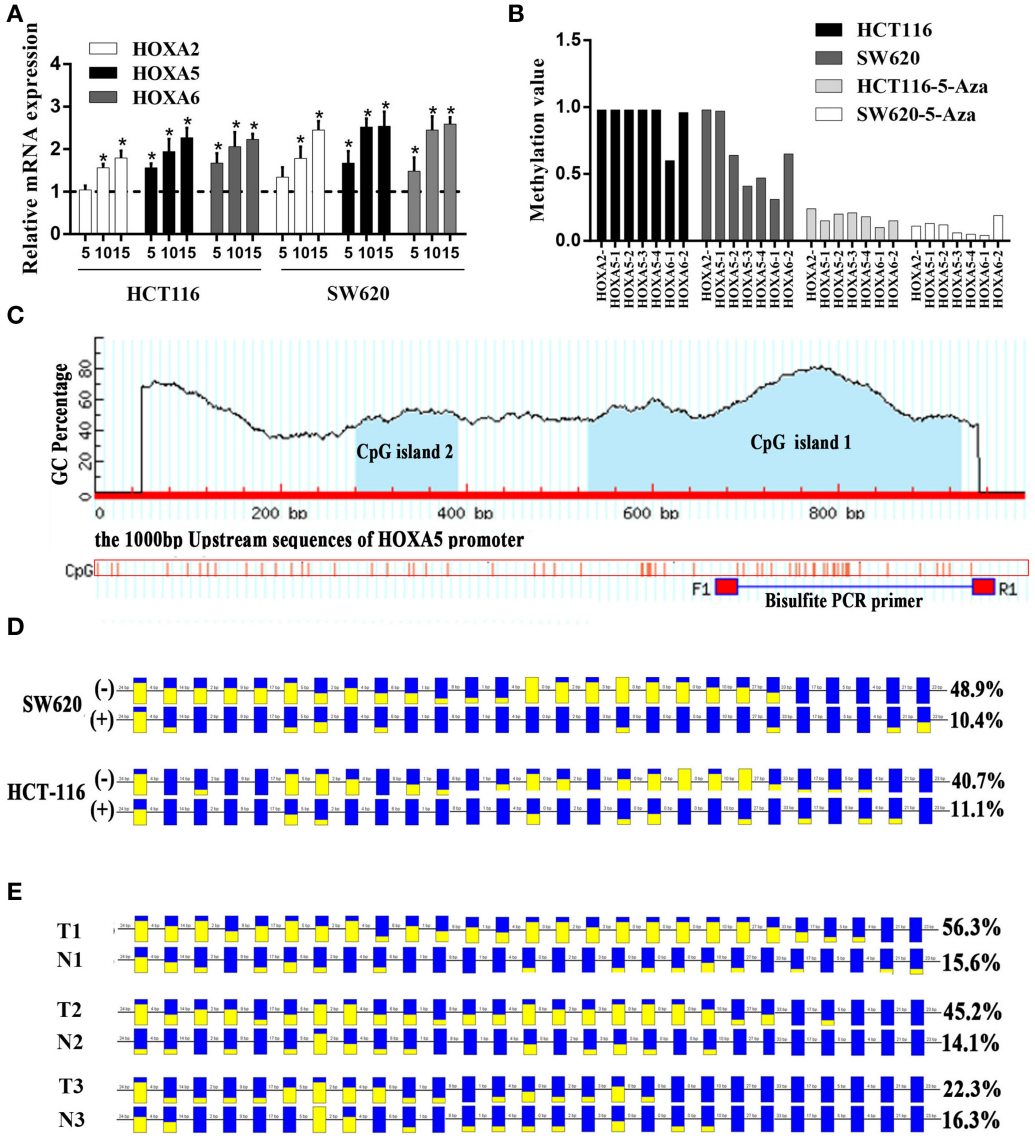
Promoter methylation responsible for the silencing of HOXA genes in colorectal cancer. **(A)** The relative expression of HOXA5 with (+) (5, 10, and 15 μmol/l) and without (–) 5-Aza treatment (set to 1) in colorectal cancer cells (Unpaired *t*-test). ^*^ indicates *p* < 0.05. **(B)** One CpG region from the CpG islands of HOXA2, 4 from HOXA5, and 2 from HOXA6 were sequenced in CRC cells with (+) (10μmol/l) and without (–) 5-Aza treatment. **(C)** MethPrimer (http://www.urogene.org/methprimer/index.html) predicts 2 CpG islands on 1,000 bp upstream promoter sequence of HOXA5 and design primers for Bisulfite-Sequencing PCR. **(D)** The result of BSP in colorectal cells SW620 and HCT-116 with (+) and without (–) 5-Aza treatment (10 umol/l). The data analyzed by BIQ Analyzer. Each square represents a CpG site. Yellow squares represent methylated CpG dinucleotides whereas blue squares represent unmethylated CpG sites. The ratios of the filled area in the squares represent the methylation status in the CpG sites. The number of base pairs between each CpG dinucleotide is indicated at the top. **(E)** The result of BSP in selected colorectal tissues including T1 and N1, T2 and N2 and T3 and N3. T: cancer tissue, N: adjacent normal tissue.

A large number of studies have shown that DNA promoter hypermethylation has critical roles in suppressing gene expression (17). Considering that the HOXA5, HOXA2, and HOXA6 promoters are very frequently hypermethylated, we speculated that these three genes would be downregulated in CRC tissues. A previous study (18) reported a statistically significant downregulation of HOXA5 expression in carcinoma relative to normal colon tissue. Similar to this result, downregulation was also observed for the HOXA2 and HOXA6 genes in large-scale human colorectal cancer expression databases. Notably, the downregulation of these three genes mainly occurs in early tumor stages including adenoma and CRC tissue without metastasis (Figures 5A–C). This agrees with the findings discussed above that the highest methylation of the HOXA genes occurs in the early stages of colorectal cancer tissue. To further verifies these conclusions, 15 paired colorectal cancer tissues and adjacent tissues were analyzed with RT-qPCR. We observed an identical pattern in that the HOXA genes were further decreased in tumor tissues without metastasis (Figure 5D). Additionally, the correlation analysis in TCGA and our colorectal cancer tissues showed a significant negative correlation between methylation levels and the expression of HOXA5 and HOXA6 genes. No identical negative correlation between HOXA2 expression and methylation was observed in the selected 372 TCGA samples and our tissues (Figures 5E–G). All indicated promoter methylation may be responsible for the silencing of 3 HOXA genes in colorectal cancer, especially the HOXA5 and HOXA6. To further confirm this conclusion, we treated 2 CRC cell lines with the demethylation agent 5-Aza and found that the expression of 3 HOXA genes was significantly upregulated (Figure 6A) and the promoter regions were significantly demethylated by targeted bisulfite sequencing (Figure 6B). Additionally, in order to further ensure the reliability of the results of MethylTarget TM assays (targeted bisulfite sequencing) developed by Genesky BioTech (Shanghai, China). DNA methylation analysis of HOXA5 gene was selected for a classical experiment-BSP. We predicted that there were two CpG islands at 1,000 bp upstream of the HOXA5 promoter, with sizes of 401 bp (CpG island 1: start-end, 67–467), and 108 bp (CpG island 2: start-end, 610–717) (Figure 6C). Given that island 1 is closer to the transcription initiation site, its BSP assay was selected and the results showed identical result that the promoter region was significantly demethylated when cells treated with the demethylation reagent 5-Aza (Figure 6D). Moreover, three paired colorectal tissues also selected for BSP. We found that tissue 1 and 2 exhibiting lower HOXA5 expression than corresponding non-cancerous tissues revealed a stronger methylation pattern, whereas case 3 with medium HOXA5 expression revealed an identical pattern (Figure 6E). These results further confirmed the conclusion discussed above.

## Discussion

In this study, we tried to characterize genes regulated by hypermethylation in colorectal cancer. We first used data from COSMIC (14), an expert-curated database of somatic mutations in human cancer, combined with literature curation and high-quality sources such as TCGA and The International Cancer Genome Consortium (ICGC). The results identified the top 20 genes with extremely high frequencies of hypermethylation in CRC. These genes included HOXA5, PCDHGC3, HOXA2, MAP7D1, ANGPT2, EPHB3, FOSB, GPC6, MSX1, PRDM13, NR5A2, TBR1, FMN2, C19orf26, CLIC6, HLA-DPB1, HOXA6, IRS4, CHD9, and ANKRD13B. Notably, only four of these genes, PCDHGC3 (19), EPHB3 (20), GPC6 (21), and NR5A2 (22), have been reported as aberrantly methylated in CRC. Whether methylation of the other 16 genes is involved in colorectal tumorigenesis needs further study.

What is particularly intriguing is that there are 3 HOXA genes in the top 20 genes, including HOXA5, HOXA2, and HOXA6. A previous study revealed that HOX genes, which code for proteins that function as important master regulatory transcription factors during embryogenesis, have a critical role in the development of human cancer (23). HOXA5, 2, and 6 are methylation targets associated with the progression of many cancers; for example, increased methylation in the HOXA5 promoter region is associated with its decreased expression during skin tumor progression (24), and aberrantly hypermethylated HOXA2 represses metalloproteinase-9 through TBP and promotes invasion in nasopharyngeal carcinoma (25). Surprisingly, although we have definitively found that the methylation frequency of these three genes ranks highest in colorectal cancer compared to all other human tissues, no studies have reported the relationship between HOXA gene methylation variation and colorectal cancer.

Despite a lack of studies on methylation, HOXA5 has been implicated in colorectal cancer. Ordóñez-Morán found that HOXA5 is significantly downregulated and counteracts stem cell traits by inhibiting wnt signaling in colorectal cancer (18). A similar pattern was observed for HOXA2 and HOXA6 expression in other human tumors (25, 26), although there are no reports on either gene in CRC. Considering that colorectal tissues have decreased HOXA5 expression and an extremely high frequency of methylation, we postulate that hypermethylation of HOXA5 leads to its silencing. Extensive research has demonstrated that DNA methylation at gene promoter CpG islands is correlated with permanent expression silencing (9). To address this issue, we analyzed the correlation of the HOXA5 methylation level and expression. The results showed a strong inverse correlation between the expression and methylation level of HOXA5. In addition, demethylation treatment can reactivate the expression of HOXA5. Together, this suggests that the downregulation of HOXA5 expression is caused by hypermethylation. An identical pattern was observed in HOXA6. No identical negative correlation between HOXA2 expression and methylation was observed in selected TCGA tissues and our tissues. The reason for this is unclear. In the future, more clinical samples may need to be collected to prove this conclusion.

As mentioned above, HOXA5, HOXA2, and HOXA6 are frequently methylated, and this leads to silencing of these genes and progression of colorectal cancer. We wanted to test whether the hypermethylation of the HOXA genes correlated with the clinicopathological features of CRC. We found that compared with normal tissues, HOXA2, HOXA5, and HOXA6 showed significantly high methylation levels. The HOXA ROC curves had excellent diagnostic ability to distinguish tissues from healthy individuals and tumor patients. Furthermore, the highest methylation of HOXA5 and HOXA2 was detected in the early stages of colorectal cancer including stage I, N0, MO, and no perineural invasion. The methylation levels declined as tumors progressed. Together, these findings indicate that HOXA methylation is an early event in CRC. Identical patterns have been observed in non-small cell lung cancers (NSCLCs). Kim DS (27) found that there was no correlation between HOXA5 methylation and survival in all NSCLCs or at stages II-IV. However, for patients with stage I disease, HOXA5 methylation was associated with a slightly worse survival rate (*P* = 0.09). Apart from the HOXA genes, previous research has identified DNA hypermethylation as an early event in tumorigenesis, most likely playing a critical role in cancer initiation and providing a fertile ground for the accumulation of overwhelmingly simultaneous genetic and epigenetic mutations (9, 28).

As *Inra JA* and *Syngal S*. reviewed in “Colorectal cancer in young adults” (29), the incidence of colorectal cancer in young people has been increasing over the past few decades when compared with patients over the age of 50. According to the prediction model, the incidence of colorectal cancer will continue to increase among people < 50 years old in 2030. There will be one colon cancer diagnosis in every 10 individuals, and there will be one rectal cancer occurrence in every four people. In our article, we found that HOXA5 and HOXA6 were more likely to be methylated in patients < 60 years of age, so these two genes may serve as screening indexes for younger cancer patients. In our study, we also evaluated the association between the methylation patterns of HOXA5, HOXA2, and HOXA6 and CIMP or MSI status, the result found that promoter DNA hypermethylation of HOXA5, HOXA2, and HOXA6 is associated with MSI-L, MSS, CIMP.L, and non-CIMP tumors, especially in MSS and non-CIMP samples. Previous study has confirmed that MSI-H tumor had a decreased likelihood to metastasize, and was a favorable prognostic marker in patients with stage II disease (30). Considering that 3 HOXA genes showed low methylation value in MSI-H, we think that 3 HOXA gene may promote the progression of CRC, and Huelsken J etc. (18) have identified that HOXA5 can play this role. But, compared with MSI-H tumor, those patients characterized as MSI-L or MSS had improved outcome with 5-FU adjuvant therapy (31), which suggest that hypermethylation of 3 HOXA gens may have a benefit impact of adjuvant therapy. An identical pattern had been found in CIMP analysis, the tumor with CIMP.L and non-CIMP presented higher methylation value, which analogous to the fact that CpG island methylator phenotype (CIMP) was associate with microsatellite instability (32, 33).

More importantly, the use of the DNA methylation pattern of a particular gene or a set of selected genes for clinical assessment is perfectly rationale because of its irreplaceable advantages, such as its frequency, stability, and variability between different patients. Moreover, DNA for methylation analysis is less prone to degradation than RNA, and DNA can be stably obtained from fluids, feces, blood and fine needle aspirates of primary tumors (9). In our study, the methylation of the HOXA genes is stable not only in the TCGA-CRC cohort but also in our CRC cohort. In addition, unlike genetic modifications, epigenetic changes do not change the primary DNA sequences and are reversible, making them a potential therapeutic target (28). In our study, we have found that the expression of silenced HOXA genes in CRC could be restored by treating with the demethylating agent 5-aza-20-deoxycytidine.

Additionally, although the highest methylation of HOXA5, HOXA2, and HOXA6 occurs in early stages of colorectal cancer tissues, methylation level at any stage of the tumor was still significantly higher than in normal tissues, which indicate HOXA5, HOXA2, and HOXA6 may be involved in the progression of colorectal cancer. Previously study (18) has found that in colorectal cancer, HOXA5 is downregulated, and its re-expression can induce loss of the cancer stem cell phenotype, preventing tumor progression, and metastasis. High expression levels of HOXA5 can identify patients with an improved probability of relapse-free survival. It is noteworthy that some researchers have found that the expression of HOXA6 in CRC tumor tissue was higher than that in adjacent normal tissue and HOXA6 can serve as a tumor promoter in CRC (34), in our study we only found HOXA6 methylation was associated with age, KRAS mutation and its downregulation in early stage CRC. So, more research is needed in future to confirm the true role of HOXA6 in colorectal cancer. In this article, we first analyzed the reasons for the abnormal expression of the 3 HOXA genes in colorectal cancer, which may offer promising therapeutic approaches for CRC.

Our results suggest that the high frequency of hypermethylation of HOXA2, HOXA5, and HOXA6 in the early stages of CRC was important events in CRC. HOXA hypermethylation is negatively associated with the expression of these genes, especially in early stages of CRC. Considering that many CRC patients present with advanced disease, early detection can lead to reduced mortality. Therefore, developing a HOXA gene methylation assay as a diagnostic tool for early detection of CRC, especially HOXA2 and HOXA5, would have substantial clinical benefits.

## Ethics Statement

The study was approved by the Ethics Committee of Central South University. All subjects gave written informed consent in accordance with the declaration of Helsinki.

## Author Contributions

DL, YB, and ZF mainly finished experiments, performed statistical analysis, and draft the manuscript. WL and CY finished experiments and participated in data analysis. YG, CL, and YZ mainly collected tumor tissues and performed clinical pathology data statistics. QH and GH contributed in designing the study. XL contributed in funding, designing the study and coordination.

### Conflict of Interest Statement

The authors declare that the research was conducted in the absence of any commercial or financial relationships that could be construed as a potential conflict of interest.

## Supplementary Material

Supplemental Figure 1 ROC results for HOXA2 and HOXA5 in distinguishing tissues from healthy individuals and Stage I patients. **(A,B)** The receiver operator characteristic curves were used to assess the clinical diagnostic utility of DNA methylation of HOXA2 and HOXA5 in distinguishing tissues from healthy individuals and stage I patients.

Supplemental Table 1 Clinical material used in this article.

Supplemental Table 2 Methylation level of each CG site in the CpG island regions of HOXA5 and HOXA6 gene in 9 paired colorectal adenocarcinoma samples and adjacent non-tumor colonic tissue samples.

Supplemental Table 3 The relationship between the methylation levels of HOXA5 and clinicopathological data.

Supplemental Table 4 The relationship between the methylation levels of HOXA6 and clinicopathological data.

Supplemental Table 5 The MSI status and the methylation value of HOXA5, HOXA2, and HOXA6.

Supplemental Table 6 The methylation_subtype status and the methylation value of HOXA5, HOXA2, and HOXA6.
