# Supplementary figures and images for: Study of Promoter Methylation Patterns of HOXA2, HOXA5, and HOXA6 and Its Clinicopathological Characteristics in Colorectal Cancer
Source: Front Oncol. 2019 May 21;9:394. doi: 10.3389/fonc.2019.00394 (PMC6536611; doi:10.3389/fonc.2019.00394)

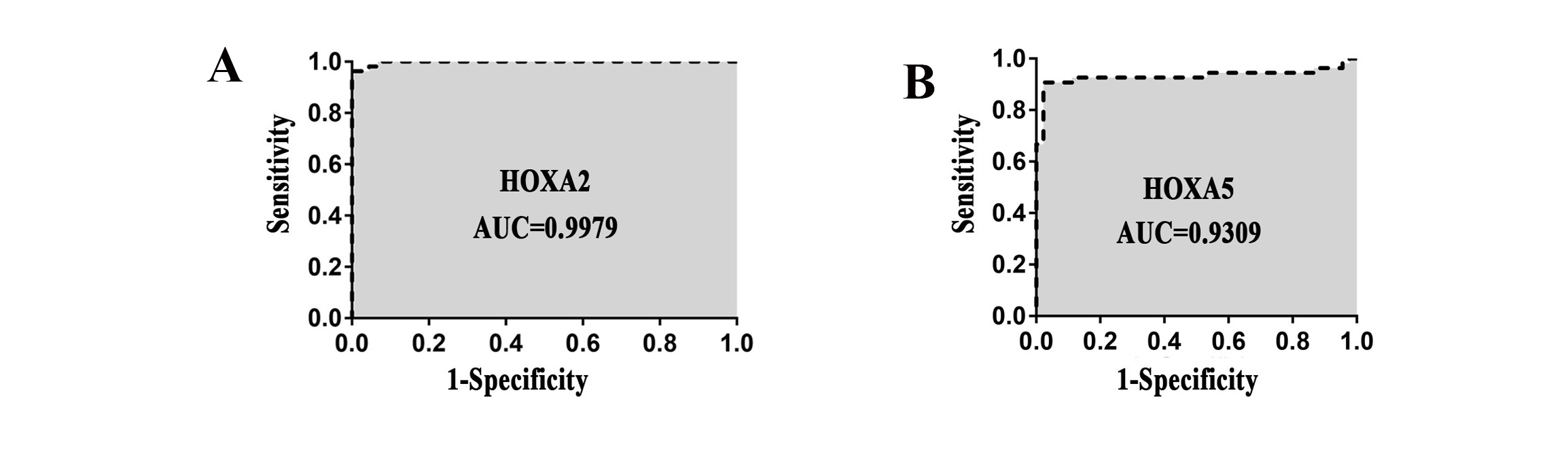

Supplement: Supplemental Figure 1 — ROC results for HOXA2 and HOXA5 in distinguishing tissues from healthy individuals and Stage I patients. (A,B) The receiver operator characteristic curves were used to assess the clinical diagnostic utility of DNA methylation of HOXA2 and HOXA5 in distinguishing tissues from healthy individuals and stage I patients. [file Image_1.JPEG]
