# Supplementary material for: Study of Promoter Methylation Patterns of HOXA2, HOXA5, and HOXA6 and Its Clinicopathological Characteristics in Colorectal Cancer
Source: Front Oncol. 2019 May 21;9:394. doi: 10.3389/fonc.2019.00394 (PMC6536611; doi:10.3389/fonc.2019.00394)
Supplement: Supplemental Table 1 — Clinical material used in this article. [file Table_1.DOCX]

| **Supplemental table 1. Clinical material used in this article** | | | | | | | | |
| --- | --- | --- | --- | --- | --- | --- | --- | --- |
| **No.** | **Sex** | **Age** | **lymph node** | **TMN** | **Pathology** | **Differentiation** | **MSI** | **Organ** |
| 1 | M | 67 | 5(14) | T4aN2aM0 | Adenocarcinoma | moderate | no | rectum |
| 2 | M | 53 | 0(22) | T2N0M0 | Adenocarcinoma | moderate | - | rectum |
| 3 | M | 72 | 4(20) | T4aN2aM0 | Adenocarcinoma | poor | - | Right colon |
| 4 | M | 52 | 0(16) | T3N0M0 | Adenocarcinoma | moderate | - | rectum |
| 5 | M | 52 | 3(25) | T3N1bM0 | Adenocarcinoma | moderate | - | Sigmoid colon |
| 6 | F | 57 | 0(15) | T4aN0M0 | Adenocarcinoma | moderate | - | Left colon |
| 7 | F | 51 | 0(14) | T4aN0M0 | Adenocarcinoma | moderate | no | rectum |
| 8 | F | 66 | 19(27) | T3N2bM0 | Adenocarcinoma | poor | no | rectum |
| 9 | F | 71 | 0(16) | T4bN0M1b | Adenocarcinoma | moderate | no | rectum |
| 10 | M | 45 | 7 (23) | T4aN2bM1b | Adenocarcinoma | moderate | - | Right colon |
| 11 | M | 68 | 5(18) | T4aN2aM1a | Adenocarcinoma | moderate | - | Right colon |
| 12 | F | 87 | 4(25) | T4bN2aM1a | Adenocarcinoma | poor | no | Right colon |
| 13 | F | 57 | 0(15) | T3N1cM1a | Adenocarcinoma | moderate | no | Left colon |
| 14 | M | 69 | 0(21) | T1NxM0 | Adenocarcinoma | poor | - | Right colon |
| 15 | F | 67 | 0(24) | T2N0M0 | Adenocarcinoma | moderate | - | Right colon |
| No.1-9 were selected for MethylTarget assays, all tissues were selected for qRT-PCR. | | | | | | | | |
