# Supplementary material for: Study of Promoter Methylation Patterns of HOXA2, HOXA5, and HOXA6 and Its Clinicopathological Characteristics in Colorectal Cancer
Source: Front Oncol. 2019 May 21;9:394. doi: 10.3389/fonc.2019.00394 (PMC6536611; doi:10.3389/fonc.2019.00394)
Supplement: Supplemental Table 2 — Methylation level of each CG site in the CpG island regions of HOXA5 and HOXA6 gene in 9 paired colorectal adenocarcinoma samples and adjacent non-tumor colonic tissue samples. [file Table_2.DOCX]

| **Supplemental tabel 2: Methylation level of each CG site in the CpG island regions of HOXA5 and HOXA6 gene in 9 paired colorectal adenocarcinoma samples and adjacent non-tumor colonic tissue samples.** | | | | | | | |
| --- | --- | --- | --- | --- | --- | --- | --- |
| Target | Position | Type | P-value(Ttest) | OR(L95-U95)(Logistic) | MethylDiff | C mean | N mean |
| HOXA5_1 | 31 | CG | 0.40836 | 1.0270(0.9668-1.0910) | 0.06702788 | 0.705629 | 0.638601 |
| HOXA5_1 | 42 | CG | 0.088003 | 1.0610(0.9887-1.1386) | 0.13112284 | 0.633261 | 0.502138 |
| HOXA5_1 | 45 | CG | 0.2613 | 1.0371(0.9745-1.1036) | 0.09187746 | 0.713554 | 0.621676 |
| HOXA5_1 | 54 | CG | 0.31434 | 1.0318(0.9725-1.0948) | 0.0854898 | 0.76616 | 0.68067 |
| HOXA5_1 | 65 | CG | 0.178359 | 1.0462(0.9798-1.1172) | 0.10620652 | 0.678941 | 0.572734 |
| HOXA5_1 | 87 | CG | 0.145421 | 1.0473(0.9837-1.1150) | 0.12195295 | 0.702431 | 0.580478 |
| HOXA5_1 | 111 | CG | 0.022379 | 1.1024(1.0001-1.2151) | 0.1538287 | 0.597389 | 0.443561 |
| HOXA5_1 | 125 | CG | 0.034062 | 1.0807(0.9970-1.1715) | 0.1636031 | 0.684722 | 0.521119 |
| HOXA5_1 | 136 | CG | 0.21078 | 1.0420(0.9775-1.1109) | 0.10130286 | 0.761123 | 0.65982 |
| HOXA5_1 | 139 | CG | 0.127557 | 1.0508(0.9847-1.1214) | 0.12549378 | 0.736628 | 0.611134 |
| HOXA5_1 | 142 | CG | 0.109098 | 1.0528(0.9869-1.1231) | 0.13355121 | 0.751097 | 0.617546 |
| HOXA5_1 | 144 | CG | 0.135317 | 1.0515(0.9832-1.1246) | 0.1199187 | 0.737578 | 0.61766 |
| HOXA5_1 | 148 | CG | 0.164081 | 1.0455(0.9818-1.1132) | 0.11590345 | 0.753228 | 0.637325 |
| HOXA5_1 | 154 | CG | 0.198339 | 1.0412(0.9796-1.1067) | 0.10881093 | 0.764263 | 0.655452 |
| HOXA5_1 | 167 | CG | 0.27766 | 1.0355(0.9738-1.1011) | 0.08923239 | 0.742973 | 0.653741 |
| HOXA5_1 | 175 | CG | 0.075116 | 1.0633(0.9905-1.1415) | 0.13888454 | 0.706052 | 0.567167 |
| HOXA5_1 | 181 | CG | 0.0192577 | 1.1028(1.0017-1.2141) | 0.16219811 | 0.605107 | 0.442909 |
| HOXA5_2 | 35 | CG | 0.105619 | 1.0579(0.9864-1.1346) | 0.12416199 | 0.598461 | 0.474299 |
| HOXA5_2 | 39 | CG | 0.038303 | 1.0749(0.9976-1.1582) | 0.16148784 | 0.594954 | 0.433466 |
| HOXA5_2 | 55 | CG | 0.159017 | 1.0512(0.9805-1.1271) | 0.10487546 | 0.614278 | 0.509403 |
| HOXA5_2 | 61 | CG | 0.07358 | 1.0660(0.9905-1.1473) | 0.13416318 | 0.548472 | 0.414308 |
| HOXA5_2 | 107 | CG | 0.39554 | 1.0321(0.9630-1.1062) | 0.05905172 | 0.618456 | 0.559404 |
| HOXA5_2 | 140 | CG | 0.47022 | 1.0276(0.9583-1.1018) | 0.04950572 | 0.678512 | 0.629007 |
| HOXA5_2 | 150 | CG | 0.42094 | 1.0290(0.9632-1.0994) | 0.0584414 | 0.694225 | 0.635784 |
| HOXA5_2 | 154 | CG | 0.41986 | 1.0289(0.9635-1.0987) | 0.05912055 | 0.704642 | 0.645522 |
| HOXA5_2 | 156 | CG | 0.317 | 1.0366(0.9687-1.1091) | 0.07242822 | 0.68362 | 0.611192 |
| HOXA5_2 | 158 | CG | 0.49396 | 1.0250(0.9589-1.0956) | 0.04906887 | 0.713625 | 0.664556 |
| HOXA5_2 | 162 | CG | 0.23216 | 1.0452(0.9737-1.1218) | 0.08419599 | 0.671081 | 0.586885 |
| HOXA5_2 | 165 | CG | 0.54978 | 1.0230(0.9539-1.0971) | 0.04072816 | 0.675882 | 0.635154 |
| HOXA5_3 | 34 | CG | 0.171541 | 1.0463(0.9814-1.1155) | 0.10683526 | 0.663594 | 0.556759 |
| HOXA5_3 | 49 | CG | 0.25326 | 1.0398(0.9746-1.1094) | 0.08613683 | 0.68516 | 0.599023 |
| HOXA5_3 | 92 | CG | 0.30518 | 1.0341(0.9723-1.0998) | 0.08097529 | 0.698351 | 0.617376 |
| HOXA5_3 | 114 | CG | 0.21472 | 1.0408(0.9786-1.1069) | 0.09949451 | 0.711539 | 0.612044 |
| HOXA5_3 | 121 | CG | 0.171125 | 1.0449(0.9819-1.1119) | 0.11039797 | 0.707877 | 0.597479 |
| HOXA5_3 | 123 | CG | 0.181765 | 1.0441(0.9809-1.1115) | 0.10717467 | 0.702601 | 0.595426 |
| HOXA5_3 | 129 | CG | 0.051195 | 1.0563(0.9958-1.1204) | 0.1904221 | 0.618015 | 0.427593 |
| HOXA5_3 | 131 | CG | 0.182784 | 1.0436(0.9809-1.1103) | 0.10804983 | 0.699401 | 0.591352 |
| HOXA5_3 | 135 | CG | 0.107697 | 1.0494(0.9887-1.1137) | 0.13994721 | 0.696074 | 0.556127 |
| HOXA5_3 | 137 | CG | 0.152277 | 1.0443(0.9843-1.1080) | 0.12306448 | 0.702923 | 0.579858 |
| HOXA5_3 | 185 | CG | 0.3043 | 1.0344(0.9722-1.1005) | 0.08068166 | 0.738888 | 0.658206 |
| HOXA5_3 | 213 | CG | 0.23564 | 1.0384(0.9773-1.1032) | 0.09683177 | 0.740389 | 0.643557 |
| HOXA5_3 | 217 | CG | 0.30596 | 1.0357(0.9709-1.1048) | 0.07728344 | 0.743062 | 0.665779 |
| HOXA5_3 | 220 | CG | 0.29142 | 1.0351(0.9732-1.1009) | 0.08323291 | 0.732089 | 0.648856 |
| HOXA5_3 | 223 | CG | 0.105782 | 1.0434(0.9898-1.1000) | 0.16471063 | 0.702178 | 0.537468 |
| HOXA5_3 | 229 | CG | 0.2677 | 1.0378(0.9740-1.1058) | 0.08567688 | 0.715272 | 0.629595 |
| HOXA5_3 | 234 | CG | 0.37012 | 1.0307(0.9678-1.0976) | 0.06864414 | 0.741417 | 0.672773 |
| HOXA5_3 | 241 | CG | 0.31124 | 1.0340(0.9717-1.1002) | 0.07943788 | 0.750888 | 0.671451 |
| HOXA5_3 | 247 | CG | 0.32788 | 1.0341(0.9697-1.1027) | 0.07386966 | 0.744735 | 0.670866 |
| HOXA5_4 | 39 | CG | 0.43912 | 1.0230(0.9687-1.0803) | 0.06810195 | 0.606598 | 0.538496 |
| HOXA5_4 | 49 | CG | 0.36888 | 1.0266(0.9720-1.0844) | 0.07955861 | 0.626606 | 0.547048 |
| HOXA5_4 | 51 | CG | 0.41318 | 1.0243(0.9698-1.0819) | 0.07203422 | 0.632053 | 0.560019 |
| HOXA5_4 | 57 | CG | 0.40196 | 1.0271(0.9677-1.0900) | 0.0684265 | 0.532259 | 0.463833 |
| HOXA5_4 | 71 | CG | 0.3453 | 1.0295(0.9716-1.0909) | 0.07921534 | 0.60654 | 0.527325 |
| HOXA5_4 | 80 | CG | 0.43468 | 1.0235(0.9685-1.0815) | 0.06803625 | 0.632347 | 0.564311 |
| HOXA5_4 | 110 | CG | 0.31122 | 1.0339(0.9718-1.0999) | 0.07953928 | 0.565862 | 0.486323 |
| HOXA5_4 | 117 | CG | 0.47702 | 1.0225(0.9650-1.0833) | 0.05871264 | 0.608935 | 0.550223 |
| HOXA5_4 | 128 | CG | 0.39766 | 1.0257(0.9699-1.0847) | 0.07287786 | 0.63566 | 0.562782 |
| HOXA5_4 | 131 | CG | 0.3955 | 1.0256(0.9703-1.0840) | 0.07402895 | 0.644569 | 0.57054 |
| HOXA5_4 | 154 | CG | 0.40636 | 1.0243(0.9706-1.0811) | 0.07412841 | 0.640243 | 0.566115 |
| HOXA5_4 | 168 | CG | 0.40092 | 1.0251(0.9702-1.0830) | 0.07369615 | 0.626381 | 0.552685 |
| HOXA5_4 | 187 | CG | 0.75992 | 1.0061(0.9701-1.0435) | 0.03984009 | 0.205447 | 0.165607 |
| HOXA5_4 | 212 | CG | 0.43692 | 1.0244(0.9670-1.0852) | 0.06484918 | 0.565416 | 0.500567 |
| HOXA5_4 | 217 | CG | 0.40476 | 1.0266(0.9681-1.0886) | 0.06843357 | 0.624198 | 0.555764 |
|  | | | | | | | |
| HOXA6_1 | 59 | CG | 0.105361 | 1.1067(0.9548-1.2827) | 0.12487744 | 0.201295 | 0.076418 |
| HOXA6_1 | 83 | CG | 0.120057 | 1.0942(0.9568-1.2513) | 0.12218075 | 0.205214 | 0.083033 |
| HOXA6_1 | 87 | CG | 0.156938 | 1.0761(0.9604-1.2057) | 0.11036271 | 0.178746 | 0.068383 |
| HOXA6_1 | 96 | CG | 0.123162 | 1.0940(0.9548-1.2536) | 0.12255155 | 0.199298 | 0.076746 |
| HOXA6_1 | 110 | CG | 0.182663 | 1.0741(0.9535-1.2100) | 0.10377902 | 0.179276 | 0.075497 |
| HOXA6_1 | 122 | CG | 0.142045 | 1.0817(0.9598-1.2189) | 0.11717023 | 0.189694 | 0.072524 |
| HOXA6_1 | 124 | CG | 0.140318 | 1.0829(0.9586-1.2233) | 0.11848861 | 0.189932 | 0.071443 |
| HOXA6_1 | 127 | CG | 0.143097 | 1.0776(0.9624-1.2065) | 0.11585226 | 0.204084 | 0.088232 |
| HOXA6_1 | 130 | CG | 0.130102 | 1.0845(0.9608-1.2240) | 0.11954973 | 0.200233 | 0.080684 |
| HOXA6_1 | 143 | CG | 0.148282 | 1.0761(0.9615-1.2044) | 0.11820991 | 0.199259 | 0.081049 |
| HOXA6_1 | 147 | CG | 0.151095 | 1.0746(0.9640-1.1978) | 0.10826073 | 0.166415 | 0.058154 |
| HOXA6_1 | 150 | CG | 0.130957 | 1.0860(0.9591-1.2297) | 0.12335004 | 0.201415 | 0.078065 |
| HOXA6_1 | 157 | CG | 0.122835 | 1.0921(0.9558-1.2477) | 0.12604077 | 0.197951 | 0.071911 |
| HOXA6_1 | 164 | CG | 0.115484 | 1.1023(0.9439-1.2874) | 0.13108307 | 0.183208 | 0.052125 |
| HOXA6_1 | 171 | CG | 0.124164 | 1.0967(0.9447-1.2732) | 0.12870895 | 0.182418 | 0.05371 |
| HOXA6_1 | 173 | CG | 0.138216 | 1.0850(0.9530-1.2353) | 0.12428011 | 0.179246 | 0.054966 |
| HOXA6_1 | 176 | CG | 0.160279 | 1.0709(0.9616-1.1927) | 0.11706317 | 0.180936 | 0.063873 |
| HOXA6_1 | 180 | CG | 0.145284 | 1.0797(0.9583-1.2166) | 0.11898036 | 0.185741 | 0.066761 |
| HOXA6_2 | 42 | CG | 0.23748 | 1.0383(0.9768-1.1037) | 0.09730226 | 0.39708 | 0.299778 |
| HOXA6_2 | 48 | CG | 0.42716 | 1.0249(0.9675-1.0857) | 0.06651471 | 0.498917 | 0.432402 |
| HOXA6_2 | 53 | CG | 0.73246 | 1.0110(0.9535-1.0720) | 0.02754018 | 0.534162 | 0.506622 |
| HOXA6_2 | 84 | CG | 0.7062 | 1.0130(0.9514-1.0786) | 0.02875031 | 0.259182 | 0.230431 |
| HOXA6_2 | 92 | CG | 0.109076 | 1.0612(0.9833-1.1453) | 0.12398995 | 0.399981 | 0.275991 |
| HOXA6_2 | 98 | CG | 0.109058 | 1.0668(0.9809-1.1602) | 0.11974837 | 0.376085 | 0.256336 |
| HOXA6_2 | 105 | CG | 0.34464 | 1.0346(0.9659-1.1082) | 0.07057473 | 0.395135 | 0.32456 |
| HOXA6_2 | 109 | CG | 0.130682 | 1.0610(0.9789-1.1499) | 0.11504013 | 0.325496 | 0.210456 |
| HOXA6_2 | 126 | CG | 0.078201 | 1.1314(0.9721-1.3168) | 0.11225343 | 0.22473 | 0.112477 |
| HOXA6_2 | 162 | CG | 0.051277 | 1.1013(0.9904-1.2247) | 0.1514184 | 0.391558 | 0.24014 |
| HOXA6_2 | 171 | CG | 0.087115 | 1.0827(0.9815-1.1943) | 0.13816561 | 0.342053 | 0.203888 |
| HOXA6_2 | 180 | CG | 0.194464 | 1.0573(0.9675-1.1555) | 0.10471735 | 0.261614 | 0.156897 |
| HOXA6_2 | 183 | CG | 0.07482 | 1.0911(0.9840-1.2100) | 0.14268195 | 0.351334 | 0.208652 |
| HOXA6_2 | 188 | CG | 0.059862 | 1.0842(0.9855-1.1928) | 0.16490402 | 0.358006 | 0.193102 |
| HOXA6_2 | 192 | CG | 0.06212 | 1.1041(0.9858-1.2365) | 0.15289307 | 0.311587 | 0.158694 |
| HOXA6_2 | 198 | CG | 0.079617 | 1.0819(0.9805-1.1938) | 0.15448787 | 0.310796 | 0.156308 |
| HOXA6_2 | 213 | CG | 0.086043 | 1.0654(0.9850-1.1524) | 0.15487916 | 0.398568 | 0.243689 |
| HOXA6_2 | 215 | CG | 0.081938 | 1.0743(0.9822-1.1750) | 0.15713951 | 0.339998 | 0.182859 |
