# Supplementary material for: Study of Promoter Methylation Patterns of HOXA2, HOXA5, and HOXA6 and Its Clinicopathological Characteristics in Colorectal Cancer
Source: Front Oncol. 2019 May 21;9:394. doi: 10.3389/fonc.2019.00394 (PMC6536611; doi:10.3389/fonc.2019.00394)
Supplement: Supplemental Table 3 — The relationship between the methylation levels of HOXA5 and clinicopathological data. [file Table_3.docx]

| **Supplemental table 3 the relationship between the methylation levels of HOXA5 and clinicopathological data** | | | | |
| --- | --- | --- | --- | --- |
|  |  |  |  |  |
| **Factor#** | **No.** | **HOXA5** | | ***P*** |
|  |  | **Low, *n* (%)** | **High, *n* (%)** |  |
| **Age** | | | | |
| <60 | 136 | 38 (27.94) | 98 (72.06) | 0.011* |
| ≥60 | 252 | 104 (41.27) | 148 (58.73) |  |
| **Gender** | | | | |
| male | 210 | 74 (35.24) | 136 (64.76) | 0.750 |
| female | 178 | 66 (37.08) | 112 (62.92) |  |
| **Height** | | | | |
| <170 | 138 | 55 (39.86) | 83(60.14) | 0.177 |
| ≥170 | 151 | 48 (31.79) | 103 (68.21) |  |
| **Weight** | | | | |
| <80 | 156 | 62 (39.74) | 94 (60.26) | 0.237 |
| ≥80 | 152 | 50 (32.89) | 102 (67.11) |  |
| **Race** | | | | |
| asian | 12 | 2(16.67) | 10 (83.33) | 0.292 |
| black | 62 | 25(40.32) | 37(59.68) |  |
| white | 280 | 100(35.71) | 180(64.29) |  |
| **T** | | | | |
| T1 | 11 | 2 (18.18) | 9 (81.82) | 0.023*^a^ |
| T2 | 54 | 11 (20.37) | 43 (79.63) |  |
| T3 | 270 | 108 (40.00) | 162 (60.00) |  |
| T4 | 51 | 21 (41.18) | 30 (58.82) |  |
| **N** | | | | |
| N0 | 212 | 71 (33.49) | 141 (66.51) | 0.337 |
| N1 | 103 | 43 (41.75) | 60 (58.25) |  |
| N2 | 70 | 27 (38.57) | 43 (61.43) |  |
| **M** | | | | |
| M0 | 264 | 90 (34.09) | 174 (65.91) | 0.013* |
| M1 | 53 | 28 (52.83) | 25 (47.17) |  |
| **Stage** | | | | |
| Ⅰ | 54 | 11 (20.37) | 43 (79.63) | 0.014*^a^ |
| Ⅱ | 143 | 52 (36.36) | 91 (63.64) |  |
| Ⅲ | 118 | 46 (38.98) | 72(61.02) |  |
| Ⅳ | 54 | 27 (50.00) | 27 (50.00) |  |
| **Lymphovascular invasion** |  |  |  |  |
| yes | 107 | 36 (33.64) | 71 (66.46) | 0.429 |
| no | 231 | 88(38.10) | 143(61.90) |  |
| **Vascular invasion** |  |  |  |  |
| yes | 78 | 33 (42.31) | 45 (57.69) | 0.244 |
| no | 254 | 89 (35.04) | 165 (64.96) |  |
| **Perineural Invasion** |  |  |  |  |
| yes | 59 | 26(44.07) | 33(55.93) | 0.105 |
| no | 170 | 55 (32.35) | 115(67.65) |  |
| **KRAS mutation** |  |  |  |  |
| yes | 28 | 11 (39.29) | 17 (60.71) | 0.573 |
| no | 28 | 8(28.57) | 20(71.43) |  |
| **Lymph nodes NO.HE** |  |  |  |  |
| 0 | 192 | 69（35.94） | 123（64.06） | 0.691 |
| 1-3 | 95 | 39（41.05） | 56（58.95） |  |
| ≥4 | 67 | 26（38.81） | 41（61.19） |  |
| **Lymph nodes NO.** | | | | |
| <18 | 168 | 64 (38.10) | 104 (61.90) | 0.999 |
| ≥18 | 189 | 72(38.10) | 117 (61.90) |  |
| **Tumor status** | | | | |
| tumor free | 246 | 81 (32.93) | 165 (67.07) | 0.046* |
| with tumor | 82 | 37 (45.12) | 45 (54.88) |  |
| **Tumor size** | | | | |
| <0.5 | 123 | 45(36.59) | 78(63.41) | 0.718 |
| ≥0.5 | 108 | 42(38.89) | 66(61.11) |  |
| **Surgical margin** |  |  |  |  |
| R0 | 256 | 88（34.38） | 168（65.62） | 0.068 |
| R1 | 5 | 3（60.00） | 2（40.00） |  |
| R2 | 7 | 5（71.43） | 2（28.57） |  |
| **History** |  |  |  |  |
| yes | 31 | 14(45.16) | 17(54.84) | 0.33 |
| no | 357 | 126(35.29) | 231(64.71) |  |
| **Tumor site** |  |  |  |  |
| ascending colon | 55 | 19 (34.55) | 36 (65.45) | 0.856 |
| cecum | 74 | 32(43.24) | 42 (56.76) |  |
| descending colon | 14 | 4 (28.57) | 10 (71.43) |  |
| ectosigmoid junction | 46 | 17 (36.96) | 29 (63.04) |  |
| transverse colon | 47 | 16(34.04) | 31(65.96) |  |
| rectum | 45 | 14(31.11) | 31 (68.89) |  |
| sigmoid colon | 88 | 32(36.36) | 56(63.64) |  |
| * means the result have statistically significance, #more details about explanation for Factor can be found in TCGA database. ^a^ If multiple hypotheses are tested, the Bonferroni correction was used to adjust the significance level for each individual test to a stricter one, the Bonferroni correction test each individual hypothesis at a significance level of 0.05/m. m is the number of hypotheses. | | | | |
