# Supplementary material for: Study of Promoter Methylation Patterns of HOXA2, HOXA5, and HOXA6 and Its Clinicopathological Characteristics in Colorectal Cancer
Source: Front Oncol. 2019 May 21;9:394. doi: 10.3389/fonc.2019.00394 (PMC6536611; doi:10.3389/fonc.2019.00394)
Supplement: Supplemental Table 4 — The relationship between the methylation levels of HOXA6 and clinicopathological data. [file Table_4.docx]

| **Supplemental Table 4 the relationship between the methylation levels of HOXA6 and clinicopathological data** | | | | |
| --- | --- | --- | --- | --- |
|  |  |  |  |  |
| **Factor#** | **No.** | **HOXA6** | | ***P*** |
|  |  | **Low, *n* (%)** | **High, *n* (%)** |  |
| **Age** | | | | |
| <60 | 136 | 61 (44.85) | 75(55.15) | 0.043* |
| ≥60 | 252 | 141 (55.95) | 111 (44.05) |  |
| **Gender** | | | | |
| male | 210 | 105 (50.00) | 105 (50.00) | 0.415 |
| female | 178 | 97 (54.49) | 81 (45.51) |  |
| **Height** | | | | |
| <170 | 138 | 76 (55.07) | 62 (44.93) | 0.409 |
| ≥170 | 151 | 75 (49.67) | 76 (50.33) |  |
| **Weight** | | | | |
| <80 | 156 | 83 (53.21) | 73 (46.79) | 0.909 |
| ≥80 | 152 | 79 (51.97) | 73 (48.03) |  |
| **Race** | | | | |
| asian | 12 | 6(50.00) | 6(50.00) | 0.834 |
| black | 62 | 31(50.00) | 31(50.00) |  |
| white | 280 | 151(53.93) | 129(46.07) |  |
| **T** | | | | |
| T1 | 11 | 5 (45.45) | 6 (54.55) | 0.607 |
| T2 | 54 | 28 (51.85) | 26 (48.15) |  |
| T3 | 270 | 138 (51.11) | 132 (48.89) |  |
| T4 | 51 | 31 (60.78) | 20 (39.22) |  |
| **N** | | | | |
| N0 | 212 | 112 (52.83) | 100 (47.17) | 0.897 |
| N1 | 103 | 55 (53.40) | 48 (46.60) |  |
| N2 | 70 | 35 (50.00) | 35 (50.00) |  |
| **M** | | | | |
| M0 | 264 | 143 (54.17) | 121 (45.83) | 0.881 |
| M1 | 53 | 28 (52.83) | 25 (47.17) |  |
| **Stage** | | | | |
| Ⅰ | 54 | 27 (50.00) | 27 (50.00) | 0.975 |
| Ⅱ | 143 | 76 (53.15) | 67 (46.85) |  |
| Ⅲ | 118 | 60 (50.85) | 58(49.15) |  |
| Ⅳ | 54 | 28 (51.85) | 26 (48.15) |  |
| **Lymphovascular invasion** |  |  |  |  |
| yes | 107 | 57(53.27) | 50(46.73) | 0.944 |
| no | 231 | 124(53.68) | 107(46.32) |  |
| **Vascular invasion** |  |  |  |  |
| yes | 78 | 43 (55.13) | 35 (44.87) | 0.796 |
| no | 254 | 135 (53.15) | 119(46.85) |  |
| **Perineural Invasion** |  |  |  |  |
| yes | 59 | 32(54.24) | 27(45.76) | 0.864 |
| no | 170 | 90(52.94) | 80(47.06) |  |
| **KRAS mutation** |  |  |  |  |
| yes | 28 | 21(75.00) | 7(25.00) | 0.001* |
| no | 28 | 9(32.14) | 19(67.86) |  |
| **Lymph nodes NO.HE** |  |  |  |  |
| 0 | 192 | 102（53.13） | 90（46.87） | 0.691 |
| 1-3 | 95 | 51（53.68） | 44（46.32） |  |
| ≥4 | 67 | 33（50.00） | 34（50.00） |  |
| **Lymph nodes NO.** | | | | |
| <18 | 168 | 91 (54.17) | 77(45.83) | 0.662 |
| ≥18 | 189 | 98(51.85) | 91(48.15) |  |
| **Tumor status** | | | | |
| tumor free | 246 | 128 (52.03) | 118(47.97) | 0.949 |
| with tumor | 82 | 43(52.44) | 39(47.56) |  |
| **Tumor size** | | | | |
| <0.5 | 123 | 77(62.60) | 46(37.40) | 0.508 |
| ≥0.5 | 108 | 63(58.33) | 45(41.67) |  |
| **Surgical margin** |  |  |  |  |
| R0 | 256 | 132（51.56） | 124（48.44） | 0.838 |
| R1 | 5 | 3（60.00） | 2（40.00） |  |
| R2 | 7 | 3（42.86） | 4（57.14） |  |
| **History** |  |  |  |  |
| yes | 31 | 16(51.61) | 15(48.39) | 0.958 |
| no | 357 | 186(52.10) | 171(47.90) |  |
| **Tumor site** |  |  |  |  |
| ascending colon | 55 | 44(80.00) | 11(20.00) | <0.001*^a^ |
| cecum | 74 | 56(75.68) | 18(24.32) |  |
| descending colon | 14 | 8(57.14) | 6(42.86) |  |
| ectosigmoid junction | 46 | 15(32.61) | 31(67.39) |  |
| transverse colon | 47 | 22(46.81) | 25(53.19) |  |
| rectum | 45 | 11(24.44) | 34(75.56) |  |
| sigmoid colon | 88 | 38(43.18) | 50(56.82) |  |
| * means the result have statistically significance , #more details about explanation for Factor can be found in TCGA database. ^a^ If multiple hypotheses are tested, the Bonferroni correction was used to adjust the significance level for each individual test to a stricter one, the Bonferroni correction test each individual hypothesis at a significance level of 0.05/m. m is the number of hypotheses. | | | | |
