# Supplementary material for: Study of Promoter Methylation Patterns of HOXA2, HOXA5, and HOXA6 and Its Clinicopathological Characteristics in Colorectal Cancer
Source: Front Oncol. 2019 May 21;9:394. doi: 10.3389/fonc.2019.00394 (PMC6536611; doi:10.3389/fonc.2019.00394)
Supplement: Supplemental Table 5 — The MSI status and the methylation value of HOXA5, HOXA2, and HOXA6. [file Table_5.DOCX]

| **supplement table 5. The MSI status and the methylation value of HOXA5, HOXA2 and HOXA6** | | | | |
| --- | --- | --- | --- | --- |
| **MSI_status** | PATIENT_ID | HOXA5 | HOXA2 | HOXA6 |
| MSI-H | TCGA-A6-2672 | 0.524898 | 0.628642 | 0.0373049 |
| MSI-H | TCGA-A6-2676 | 0.338764 | 0.750937 | 0.024619 |
| MSI-H | TCGA-A6-3809 | 0.711824 | 0.372868 | 0.0473518 |
| MSI-H | TCGA-AA-3516 | 0.762714 | 0.723186 | 0.0534473 |
| MSI-H | TCGA-AA-3518 | 0.892208 | 0.848288 | 0.0389957 |
| MSI-H | TCGA-AA-3525 | 0.807365 | 0.749127 | 0.4763163 |
| MSI-H | TCGA-AA-3543 | 0.339114 | 0.621474 | 0.0587196 |
| MSI-H | TCGA-AA-3554 | 0.514746 | 0.636218 | 0.0554928 |
| MSI-H | TCGA-AA-3672 | 0.265628 | 0.687236 | 0.0533477 |
| MSI-H | TCGA-AA-3710 | 0.754164 | 0.651022 | 0.0476888 |
| MSI-H | TCGA-AA-3715 | 0.726165 | 0.623913 | 0.511648 |
| MSI-H | TCGA-AA-3811 | 0.602192 | 0.824805 | 0.294221 |
| MSI-H | TCGA-AA-3815 | 0.316587 | 0.726221 | 0.0298101 |
| MSI-H | TCGA-AA-3821 | 0.204952 | 0.51594 | 0.0463689 |
| MSI-H | TCGA-AA-3833 | 0.305035 | 0.57463 | 0.040311 |
| MSI-H | TCGA-AA-3845 | 0.469013 | 0.67809 | 0.3128082 |
| MSI-H | TCGA-AA-3864 | 0.860785 | 0.791057 | 0.6541703 |
| MSI-H | TCGA-AA-3877 | 0.300811 | 0.679651 | 0.0281512 |
| MSI-H | TCGA-AA-3947 | 0.300595 | 0.814408 | 0.0552463 |
| MSI-H | TCGA-AA-3949 | 0.316399 | 0.480809 | 0.0305974 |
| MSI-H | TCGA-AA-3950 | 0.834127 | 0.629845 | 0.4381981 |
| MSI-H | TCGA-AA-3966 | 0.481831 | 0.804036 | 0.0646121 |
| MSI-H | TCGA-AA-A00A | 0.946693 | 0.816632 | 0.0532601 |
| MSI-H | TCGA-AA-A00E | 0.396227 | 0.631358 | 0.0488997 |
| MSI-H | TCGA-AA-A00J | 0.629324 | 0.863788 | 0.0209748 |
| MSI-H | TCGA-AA-A00R | 0.714246 | 0.703916 | 0.0778565 |
| MSI-H | TCGA-AA-A01P | 0.502838 | 0.407019 | 0.0923306 |
| MSI-H | TCGA-AA-A01Q | 0.947049 | 0.830415 | 0.4353697 |
| MSI-H | TCGA-AA-A01R | 0.865066 | 0.725615 | 0.0427769 |
| MSI-H | TCGA-AA-A022 | 0.58703 | 0.781624 | 0.0492122 |
| MSI-H | TCGA-AA-A02R | 0.410986 | 0.811728 | 0.05 |
| MSI-L | TCGA-A6-2683 | 0.945166 | 0.936129 | 0.8326245 |
| MSI-L | TCGA-A6-3808 | 0.841911 | 0.702792 | 0.3935799 |
| MSI-L | TCGA-AA-3517 | 0.905159 | 0.873396 | 0.7884198 |
| MSI-L | TCGA-AA-3520 | 0.287125 | 0.812662 | 0.039382 |
| MSI-L | TCGA-AA-3526 | 0.859089 | 0.790744 | 0.6676246 |
| MSI-L | TCGA-AA-3529 | 0.831226 | 0.784053 | 0.6725933 |
| MSI-L | TCGA-AA-3531 | 0.925658 | 0.921256 | 0.8352266 |
| MSI-L | TCGA-AA-3553 | 0.859807 | 0.620915 | 0.1874177 |
| MSI-L | TCGA-AA-3667 | 0.886781 | 0.82893 | 0.7282444 |
| MSI-L | TCGA-AA-3680 | 0.834999 | 0.724596 | 0.7012375 |
| MSI-L | TCGA-AA-3688 | 0.899575 | 0.871668 | 0.7680617 |
| MSI-L | TCGA-AA-3692 | 0.837405 | 0.804368 | 0.7205459 |
| MSI-L | TCGA-AA-3819 | 0.813792 | 0.704088 | 0.6427227 |
| MSI-L | TCGA-AA-3852 | 0.867728 | 0.759433 | 0.0893139 |
| MSI-L | TCGA-AA-3854 | 0.096671 | 0.688928 | 0.0315271 |
| MSI-L | TCGA-AA-3855 | 0.854726 | 0.809128 | 0.6674628 |
| MSI-L | TCGA-AA-3861 | 0.832 | 0.769197 | 0.3917711 |
| MSI-L | TCGA-AA-3866 | 0.850966 | 0.748204 | 0.6519785 |
| MSI-L | TCGA-AA-3930 | 0.847755 | 0.364822 | 0.2543292 |
| MSI-L | TCGA-AA-3941 | 0.908415 | 0.859381 | 0.0333126 |
| MSI-L | TCGA-AA-3972 | 0.887433 | 0.82404 | 0.7550228 |
| MSI-L | TCGA-AA-3973 | 0.916613 | 0.890628 | 0.835226 |
| MSI-L | TCGA-AA-3982 | 0.657565 | 0.670724 | 0.5788132 |
| MSI-L | TCGA-AA-A004 | 0.799534 | 0.690773 | 0.5659875 |
| MSI-L | TCGA-AA-A00K | 0.891189 | 0.74457 | 0.3821509 |
| MSI-L | TCGA-AA-A00N | 0.74022 | 0.67963 | 0.0789316 |
| MSI-L | TCGA-AA-A00O | 0.658347 | 0.702454 | 0.5208963 |
| MSI-L | TCGA-AA-A010 | 0.777438 | 0.781026 | 0.4012264 |
| MSI-L | TCGA-AA-A01G | 0.784272 | 0.658222 | 0.6046019 |
| MSI-L | TCGA-AA-A01S | 0.901667 | 0.8829 | 0.8190018 |
| MSI-L | TCGA-AA-A024 | 0.878918 | 0.837881 | 0.3357262 |
| MSI-L | TCGA-AA-A029 | 0.929885 | 0.770395 | 0.1437613 |
| MSI-L | TCGA-AA-A02E | 0.837441 | 0.878914 | 0.1622704 |
| MSI-L | TCGA-AA-A02W | 0.158247 | 0.795085 | 0.0342175 |
| MSI-L | TCGA-AG-3583 | 0.886619 | 0.799175 | 0.6638682 |
| MSI-L | TCGA-AG-3601 | 0.71325 | 0.906843 | 0.7204156 |
| MSI-L | TCGA-AG-4001 | 0.679144 | 0.535475 | 0.3725691 |
| MSI-L | TCGA-AG-4007 | 0.920028 | 0.83698 | 0.8043833 |
| MSS | TCGA-A6-2670 | 0.9383 | 0.822606 | 0.7235895 |
| MSS | TCGA-A6-2674 | 0.771669 | 0.602821 | 0.3628143 |
| MSS | TCGA-A6-2677 | 0.943215 | 0.93207 | 0.581486 |
| MSS | TCGA-A6-2678 | 0.83262 | 0.855306 | 0.7659894 |
| MSS | TCGA-A6-3807 | 0.850338 | 0.737026 | 0.6682663 |
| MSS | TCGA-A6-3810 | 0.894819 | 0.818813 | 0.7270786 |
| MSS | TCGA-AA-3514 | 0.882628 | 0.847183 | 0.6777716 |
| MSS | TCGA-AA-3519 | 0.881177 | 0.840652 | 0.7708522 |
| MSS | TCGA-AA-3521 | 0.469105 | 0.814705 | 0.0252501 |
| MSS | TCGA-AA-3522 | 0.61588 | 0.775818 | 0.0440619 |
| MSS | TCGA-AA-3524 | 0.895719 | 0.853276 | 0.7687486 |
| MSS | TCGA-AA-3527 | 0.822556 | 0.855461 | 0.0842703 |
| MSS | TCGA-AA-3530 | 0.699024 | 0.854501 | 0.2708565 |
| MSS | TCGA-AA-3532 | 0.865454 | 0.675753 | 0.4890865 |
| MSS | TCGA-AA-3534 | 0.898776 | 0.878602 | 0.0161027 |
| MSS | TCGA-AA-3538 | 0.493836 | 0.785384 | 0.0347264 |
| MSS | TCGA-AA-3542 | 0.66548 | 0.916553 | 0.4746757 |
| MSS | TCGA-AA-3544 | 0.889233 | 0.799572 | 0.6900845 |
| MSS | TCGA-AA-3548 | 0.529637 | 0.80689 | 0.3931776 |
| MSS | TCGA-AA-3549 | 0.251698 | 0.795706 | 0.0321927 |
| MSS | TCGA-AA-3552 | 0.398966 | 0.790401 | 0.0561727 |
| MSS | TCGA-AA-3555 | 0.874941 | 0.810789 | 0.6483003 |
| MSS | TCGA-AA-3556 | 0.874231 | 0.825265 | 0.7234874 |
| MSS | TCGA-AA-3558 | 0.935386 | 0.905115 | 0.8220461 |
| MSS | TCGA-AA-3560 | 0.741319 | 0.866348 | 0.0219997 |
| MSS | TCGA-AA-3561 | 0.1541 | 0.922806 | 0.0322693 |
| MSS | TCGA-AA-3562 | 0.88764 | 0.821681 | 0.7000646 |
| MSS | TCGA-AA-3664 | 0.121593 | 0.520128 | 0.0316241 |
| MSS | TCGA-AA-3666 | 0.14749 | 0.844846 | 0.0379835 |
| MSS | TCGA-AA-3673 | 0.819997 | 0.72528 | 0.4869397 |
| MSS | TCGA-AA-3675 | 0.884433 | 0.867961 | 0.7329621 |
| MSS | TCGA-AA-3678 | 0.762301 | 0.68087 | 0.0682654 |
| MSS | TCGA-AA-3679 | 0.848204 | 0.801721 | 0.4456919 |
| MSS | TCGA-AA-3681 | 0.18455 | 0.690851 | 0.0433827 |
| MSS | TCGA-AA-3684 | 0.599602 | 0.638374 | 0.077741 |
| MSS | TCGA-AA-3685 | 0.842728 | 0.732777 | 0.6404876 |
| MSS | TCGA-AA-3693 | 0.914732 | 0.896381 | 0.7956818 |
| MSS | TCGA-AA-3695 | 0.6701 | 0.876913 | 0.331007 |
| MSS | TCGA-AA-3696 | 0.865698 | 0.827039 | 0.7294076 |
| MSS | TCGA-AA-3812 | 0.616446 | 0.553934 | 0.4764563 |
| MSS | TCGA-AA-3814 | 0.659126 | 0.735866 | 0.4834805 |
| MSS | TCGA-AA-3818 | 0.868216 | 0.829542 | 0.7181132 |
| MSS | TCGA-AA-3831 | 0.853192 | 0.716765 | 0.5429467 |
| MSS | TCGA-AA-3837 | 0.774307 | 0.71932 | 0.5852349 |
| MSS | TCGA-AA-3842 | 0.591207 | 0.774107 | 0.0429598 |
| MSS | TCGA-AA-3844 | 0.91843 | 0.866825 | 0.5679765 |
| MSS | TCGA-AA-3846 | 0.835608 | 0.77775 | 0.640919 |
| MSS | TCGA-AA-3848 | 0.86404 | 0.818301 | 0.6980899 |
| MSS | TCGA-AA-3850 | 0.773149 | 0.694022 | 0.4457417 |
| MSS | TCGA-AA-3851 | 0.854258 | 0.763206 | 0.0959942 |
| MSS | TCGA-AA-3856 | 0.469526 | 0.641391 | 0.0433624 |
| MSS | TCGA-AA-3858 | 0.856956 | 0.805156 | 0.6904821 |
| MSS | TCGA-AA-3860 | 0.828717 | 0.785256 | 0.6621722 |
| MSS | TCGA-AA-3862 | 0.88073 | 0.773812 | 0.0792987 |
| MSS | TCGA-AA-3867 | 0.292663 | 0.544451 | 0.0229998 |
| MSS | TCGA-AA-3869 | 0.465395 | 0.815711 | 0.0381381 |
| MSS | TCGA-AA-3870 | 0.534224 | 0.738836 | 0.0232685 |
| MSS | TCGA-AA-3872 | 0.272562 | 0.719411 | 0.0425744 |
| MSS | TCGA-AA-3875 | 0.699085 | 0.683123 | 0.546302 |
| MSS | TCGA-AA-3939 | 0.90322 | 0.804493 | 0.4592961 |
| MSS | TCGA-AA-3952 | 0.871223 | 0.79392 | 0.6452152 |
| MSS | TCGA-AA-3955 | 0.908584 | 0.850689 | 0.3350117 |
| MSS | TCGA-AA-3956 | 0.74782 | 0.685521 | 0.5541962 |
| MSS | TCGA-AA-3968 | 0.876266 | 0.809466 | 0.7482732 |
| MSS | TCGA-AA-3970 | 0.858609 | 0.659384 | 0.6863693 |
| MSS | TCGA-AA-3971 | 0.785846 | 0.715121 | 0.6019004 |
| MSS | TCGA-AA-3975 | 0.860374 | 0.773367 | 0.7469986 |
| MSS | TCGA-AA-3976 | 0.902283 | 0.847534 | 0.7726297 |
| MSS | TCGA-AA-3977 | 0.826478 | 0.743881 | 0.6272024 |
| MSS | TCGA-AA-3979 | 0.922187 | 0.875159 | 0.7948986 |
| MSS | TCGA-AA-3980 | 0.904541 | 0.777834 | 0.6921484 |
| MSS | TCGA-AA-3984 | 0.866238 | 0.680924 | 0.5725497 |
| MSS | TCGA-AA-3986 | 0.8469 | 0.691966 | 0.5767843 |
| MSS | TCGA-AA-3989 | 0.850893 | 0.776543 | 0.0655382 |
| MSS | TCGA-AA-3994 | 0.895302 | 0.823778 | 0.1473059 |
| MSS | TCGA-AA-A00D | 0.749696 | 0.657424 | 0.0424252 |
| MSS | TCGA-AA-A00F | 0.774385 | 0.711115 | 0.264544 |
| MSS | TCGA-AA-A00L | 0.864589 | 0.891113 | 0.7960128 |
| MSS | TCGA-AA-A00Q | 0.851749 | 0.785966 | 0.3265306 |
| MSS | TCGA-AA-A00U | 0.893371 | 0.830648 | 0.6863805 |
| MSS | TCGA-AA-A00W | 0.881259 | 0.804576 | 0.4208158 |
| MSS | TCGA-AA-A00Z | 0.783705 | 0.74445 | 0.6234943 |
| MSS | TCGA-AA-A017 | 0.838575 | 0.715205 | 0.5832627 |
| MSS | TCGA-AA-A01C | 0.855417 | 0.788894 | 0.6472635 |
| MSS | TCGA-AA-A01D | 0.284128 | 0.580106 | 0.0759651 |
| MSS | TCGA-AA-A01F | 0.612167 | 0.91807 | 0.0102813 |
| MSS | TCGA-AA-A01I | 0.863022 | 0.742678 | 0.6196348 |
| MSS | TCGA-AA-A01K | 0.808829 | 0.673605 | 0.5172533 |
| MSS | TCGA-AA-A01T | 0.867319 | 0.819753 | 0.7635939 |
| MSS | TCGA-AA-A01V | 0.634487 | 0.83404 | 0.090697 |
| MSS | TCGA-AA-A01X | 0.855069 | 0.808711 | 0.697709 |
| MSS | TCGA-AA-A01Z | 0.860295 | 0.860826 | 0.0444896 |
| MSS | TCGA-AA-A02F | 0.909505 | 0.861803 | 0.6981476 |
| MSS | TCGA-AA-A02H | 0.84144 | 0.818244 | 0.6897661 |
| MSS | TCGA-AA-A02J | 0.879571 | 0.79727 | 0.6986159 |
| MSS | TCGA-AA-A02K | 0.839407 | 0.743608 | 0.6524165 |
| MSS | TCGA-AA-A02O | 0.894968 | 0.872259 | 0.7825832 |
| MSS | TCGA-AA-A02Y | 0.729055 | 0.844909 | 0.0467016 |
| MSS | TCGA-AA-A03F | 0.485482 | 0.810515 | 0.0754796 |
| MSS | TCGA-AA-A03J | 0.819876 | 0.708634 | 0.4311044 |
| MSS | TCGA-AF-2689 | 0.61264 | 0.856744 | 0.213225 |
| MSS | TCGA-AF-2691 | 0.876295 | 0.834919 | 0.4009024 |
| MSS | TCGA-AF-2692 | 0.887304 | 0.827043 | 0.0777989 |
| MSS | TCGA-AF-3400 | 0.640448 | 0.581668 | 0.041139 |
| MSS | TCGA-AF-3913 | 0.808769 | 0.851349 | 0.1626152 |
| MSS | TCGA-AG-3574 | 0.9304 | 0.882619 | 0.7801093 |
| MSS | TCGA-AG-3575 | 0.933091 | 0.833611 | 0.0506785 |
| MSS | TCGA-AG-3578 | 0.94643 | 0.863481 | 0.0279873 |
| MSS | TCGA-AG-3580 | 0.943984 | 0.770821 | 0.2636812 |
| MSS | TCGA-AG-3581 | 0.883494 | 0.801307 | 0.0246 |
| MSS | TCGA-AG-3582 | 0.949938 | 0.886047 | 0.3079346 |
| MSS | TCGA-AG-3584 | 0.917738 | 0.604282 | 0.742655 |
| MSS | TCGA-AG-3586 | 0.861949 | 0.818566 | 0.1074503 |
| MSS | TCGA-AG-3587 | 0.307902 | 0.915832 | 0.3973554 |
| MSS | TCGA-AG-3593 | 0.942628 | 0.890665 | 0.6518728 |
| MSS | TCGA-AG-3594 | 0.352756 | 0.758688 | 0.0369748 |
| MSS | TCGA-AG-3598 | 0.177268 | 0.810807 | 0.1440816 |
| MSS | TCGA-AG-3599 | 0.912978 | 0.845274 | 0.055636 |
| MSS | TCGA-AG-3600 | 0.95819 | 0.878619 | 0.5589302 |
| MSS | TCGA-AG-3602 | 0.668692 | 0.752576 | 0.4407547 |
| MSS | TCGA-AG-3605 | 0.283391 | 0.75301 | 0.0364749 |
| MSS | TCGA-AG-3609 | 0.905228 | 0.828731 | 0.7140792 |
| MSS | TCGA-AG-3611 | 0.664033 | 0.907826 | 0.0270679 |
| MSS | TCGA-AG-3612 | 0.869869 | 0.863729 | 0.0566968 |
| MSS | TCGA-AG-3726 | 0.583427 | 0.512669 | 0.3951475 |
| MSS | TCGA-AG-3727 | 0.86182 | 0.825982 | 0.5607948 |
| MSS | TCGA-AG-3728 | 0.868917 | 0.797804 | 0.5250956 |
| MSS | TCGA-AG-3878 | 0.810073 | 0.708474 | 0.5389566 |
| MSS | TCGA-AG-3881 | 0.60067 | 0.736803 | 0.0320938 |
| MSS | TCGA-AG-3882 | 0.867719 | 0.740816 | 0.6288627 |
| MSS | TCGA-AG-3883 | 0.799559 | 0.737217 | 0.5873837 |
| MSS | TCGA-AG-3885 | 0.42305 | 0.87093 | 0.0207804 |
| MSS | TCGA-AG-3887 | 0.858682 | 0.840013 | 0.7526885 |
| MSS | TCGA-AG-3890 | 0.846974 | 0.766601 | 0.1746804 |
| MSS | TCGA-AG-3892 | 0.770523 | 0.686445 | 0.4925811 |
| MSS | TCGA-AG-3893 | 0.924616 | 0.835874 | 0.5255477 |
| MSS | TCGA-AG-3894 | 0.884457 | 0.819733 | 0.5400687 |
| MSS | TCGA-AG-3896 | 0.802404 | 0.790912 | 0.4197172 |
| MSS | TCGA-AG-3898 | 0.778021 | 0.733107 | 0.3491458 |
| MSS | TCGA-AG-3901 | 0.785377 | 0.553092 | 0.3781299 |
| MSS | TCGA-AG-3902 | 0.495653 | 0.720849 | 0.1416053 |
| MSS | TCGA-AG-3909 | 0.758582 | 0.801792 | 0.7123192 |
| MSS | TCGA-AG-3999 | 0.225052 | 0.846021 | 0.0753458 |
| MSS | TCGA-AG-4005 | 0.534181 | 0.835085 | 0.028822 |
| MSS | TCGA-AG-4008 | 0.848971 | 0.791535 | 0.0814622 |
| MSS | TCGA-AG-4015 | 0.660401 | 0.874476 | 0.0354531 |
| MSS | TCGA-AG-A002 | 0.145796 | 0.852045 | 0.0182515 |
| MSS | TCGA-AG-A008 | 0.907922 | 0.897503 | 0.318326 |
| MSS | TCGA-AG-A00C | 0.876185 | 0.819587 | 0.6323087 |
| MSS | TCGA-AG-A00H | 0.621031 | 0.682444 | 0.2546642 |
| MSS | TCGA-AG-A00Y | 0.692546 | 0.828593 | 0.3984472 |
| MSS | TCGA-AG-A011 | 0.767617 | 0.686586 | 0.5265863 |
| MSS | TCGA-AG-A014 | 0.965628 | 0.86333 | 0.4243941 |
| MSS | TCGA-AG-A015 | 0.222283 | 0.859382 | 0.0133501 |
| MSS | TCGA-AG-A016 | 0.779629 | 0.848347 | 0.8308367 |
| MSS | TCGA-AG-A01J | 0.775982 | 0.682599 | 0.3698037 |
| MSS | TCGA-AG-A01L | 0.894403 | 0.701314 | 0.4867664 |
| MSS | TCGA-AG-A01N | 0.823483 | 0.43506 | 0.0324577 |
| MSS | TCGA-AG-A023 | 0.740112 | 0.661134 | 0.0614834 |
| MSS | TCGA-AG-A025 | 0.818167 | 0.760383 | 0.0286491 |
| MSS | TCGA-AG-A026 | 0.859157 | 0.781934 | 0.6186124 |
| MSS | TCGA-AG-A02G | 0.916532 | 0.846558 | 0.3645268 |
| MSS | TCGA-AG-A02X | 0.90489 | 0.862927 | 0.0214134 |
| MSS | TCGA-AG-A032 | 0.861213 | 0.734318 | 0.0308113 |
| MSS | TCGA-AY-4070 | 0.695944 | 0.768176 | 0.0813823 |
| MSS | TCGA-AY-4071 | 0.924587 | 0.840596 | 0.3350782 |
