# Supplementary material for: Study of Promoter Methylation Patterns of HOXA2, HOXA5, and HOXA6 and Its Clinicopathological Characteristics in Colorectal Cancer
Source: Front Oncol. 2019 May 21;9:394. doi: 10.3389/fonc.2019.00394 (PMC6536611; doi:10.3389/fonc.2019.00394)
Supplement: Supplemental Table 6 — The methylation_subtype status and the methylation value of HOXA5, HOXA2, and HOXA6. [file Table_6.DOCX]

| **supplement table 6. the methylation_subtype status and the methylation value of HOXA5, HOXA2 and HOXA6** | | | | |
| --- | --- | --- | --- | --- |
| **methylation_subtype** | PATIENT_ID | HOXA5 | HOXA2 | HOXA6 |
| CIMP.H | TCGA-A6-2672 | 0.524898 | 0.628642 | 0.037305 |
| CIMP.H | TCGA-A6-2676 | 0.338764 | 0.750937 | 0.024619 |
| CIMP.H | TCGA-A6-3809 | 0.711824 | 0.372868 | 0.047352 |
| CIMP.H | TCGA-AA-3516 | 0.762714 | 0.723186 | 0.053447 |
| CIMP.H | TCGA-AA-3518 | 0.892208 | 0.848288 | 0.038996 |
| CIMP.H | TCGA-AA-3525 | 0.807365 | 0.749127 | 0.476316 |
| CIMP.H | TCGA-AA-3543 | 0.339114 | 0.621474 | 0.05872 |
| CIMP.H | TCGA-AA-3664 | 0.121593 | 0.520128 | 0.031624 |
| CIMP.H | TCGA-AA-3672 | 0.265628 | 0.687236 | 0.053348 |
| CIMP.H | TCGA-AA-3710 | 0.754164 | 0.651022 | 0.047689 |
| CIMP.H | TCGA-AA-3715 | 0.726165 | 0.623913 | 0.511648 |
| CIMP.H | TCGA-AA-3815 | 0.316587 | 0.726221 | 0.02981 |
| CIMP.H | TCGA-AA-3821 | 0.204952 | 0.51594 | 0.046369 |
| CIMP.H | TCGA-AA-3833 | 0.305035 | 0.57463 | 0.040311 |
| CIMP.H | TCGA-AA-3837 | 0.774307 | 0.71932 | 0.585235 |
| CIMP.H | TCGA-AA-3845 | 0.469013 | 0.67809 | 0.312808 |
| CIMP.H | TCGA-AA-3870 | 0.534224 | 0.738836 | 0.023269 |
| CIMP.H | TCGA-AA-3877 | 0.300811 | 0.679651 | 0.028151 |
| CIMP.H | TCGA-AA-3941 | 0.908415 | 0.859381 | 0.033313 |
| CIMP.H | TCGA-AA-3947 | 0.300595 | 0.814408 | 0.055246 |
| CIMP.H | TCGA-AA-3949 | 0.316399 | 0.480809 | 0.030597 |
| CIMP.H | TCGA-AA-3950 | 0.834127 | 0.629845 | 0.438198 |
| CIMP.H | TCGA-AA-3994 | 0.895302 | 0.823778 | 0.147306 |
| CIMP.H | TCGA-AA-A00A | 0.946693 | 0.816632 | 0.05326 |
| CIMP.H | TCGA-AA-A00D | 0.749696 | 0.657424 | 0.042425 |
| CIMP.H | TCGA-AA-A00E | 0.396227 | 0.631358 | 0.0489 |
| CIMP.H | TCGA-AA-A00J | 0.629324 | 0.863788 | 0.020975 |
| CIMP.H | TCGA-AA-A01P | 0.502838 | 0.407019 | 0.092331 |
| CIMP.H | TCGA-AA-A022 | 0.58703 | 0.781624 | 0.049212 |
| CIMP.H | TCGA-AA-A029 | 0.929885 | 0.770395 | 0.143761 |
| CIMP.H | TCGA-AA-A02O | 0.894968 | 0.872259 | 0.782583 |
| CIMP.H | TCGA-AA-A02R | 0.410986 | 0.811728 | 0.05 |
| CIMP.H | TCGA-AA-A03F | 0.485482 | 0.810515 | 0.07548 |
| CIMP.H | TCGA-AG-3575 | 0.933091 | 0.833611 | 0.050678 |
| CIMP.H | TCGA-AG-3600 | 0.95819 | 0.878619 | 0.55893 |
| CIMP.H | TCGA-AG-A02X | 0.90489 | 0.862927 | 0.021413 |
| CIMP.L | TCGA-A6-2683 | 0.945166 | 0.936129 | 0.832625 |
| CIMP.L | TCGA-AA-3522 | 0.61588 | 0.775818 | 0.044062 |
| CIMP.L | TCGA-AA-3527 | 0.822556 | 0.855461 | 0.08427 |
| CIMP.L | TCGA-AA-3549 | 0.251698 | 0.795706 | 0.032193 |
| CIMP.L | TCGA-AA-3555 | 0.874941 | 0.810789 | 0.6483 |
| CIMP.L | TCGA-AA-3556 | 0.874231 | 0.825265 | 0.723487 |
| CIMP.L | TCGA-AA-3558 | 0.935386 | 0.905115 | 0.822046 |
| CIMP.L | TCGA-AA-3673 | 0.819997 | 0.72528 | 0.48694 |
| CIMP.L | TCGA-AA-3675 | 0.884433 | 0.867961 | 0.732962 |
| CIMP.L | TCGA-AA-3680 | 0.834999 | 0.724596 | 0.701238 |
| CIMP.L | TCGA-AA-3681 | 0.18455 | 0.690851 | 0.043383 |
| CIMP.L | TCGA-AA-3684 | 0.599602 | 0.638374 | 0.077741 |
| CIMP.L | TCGA-AA-3695 | 0.6701 | 0.876913 | 0.331007 |
| CIMP.L | TCGA-AA-3818 | 0.868216 | 0.829542 | 0.718113 |
| CIMP.L | TCGA-AA-3831 | 0.853192 | 0.716765 | 0.542947 |
| CIMP.L | TCGA-AA-3842 | 0.591207 | 0.774107 | 0.04296 |
| CIMP.L | TCGA-AA-3848 | 0.86404 | 0.818301 | 0.69809 |
| CIMP.L | TCGA-AA-3850 | 0.773149 | 0.694022 | 0.445742 |
| CIMP.L | TCGA-AA-3852 | 0.867728 | 0.759433 | 0.089314 |
| CIMP.L | TCGA-AA-3854 | 0.096671 | 0.688928 | 0.031527 |
| CIMP.L | TCGA-AA-3855 | 0.854726 | 0.809128 | 0.667463 |
| CIMP.L | TCGA-AA-3861 | 0.832 | 0.769197 | 0.391771 |
| CIMP.L | TCGA-AA-3930 | 0.847755 | 0.364822 | 0.254329 |
| CIMP.L | TCGA-AA-3966 | 0.481831 | 0.804036 | 0.064612 |
| CIMP.L | TCGA-AA-3972 | 0.887433 | 0.82404 | 0.755023 |
| CIMP.L | TCGA-AA-3979 | 0.922187 | 0.875159 | 0.794899 |
| CIMP.L | TCGA-AA-3989 | 0.850893 | 0.776543 | 0.065538 |
| CIMP.L | TCGA-AA-A004 | 0.799534 | 0.690773 | 0.565988 |
| CIMP.L | TCGA-AA-A00Q | 0.851749 | 0.785966 | 0.326531 |
| CIMP.L | TCGA-AA-A00R | 0.714246 | 0.703916 | 0.077856 |
| CIMP.L | TCGA-AA-A00U | 0.893371 | 0.830648 | 0.68638 |
| CIMP.L | TCGA-AA-A01D | 0.284128 | 0.580106 | 0.075965 |
| CIMP.L | TCGA-AA-A01G | 0.784272 | 0.658222 | 0.604602 |
| CIMP.L | TCGA-AA-A01I | 0.863022 | 0.742678 | 0.619635 |
| CIMP.L | TCGA-AA-A01Q | 0.947049 | 0.830415 | 0.43537 |
| CIMP.L | TCGA-AA-A01R | 0.865066 | 0.725615 | 0.042777 |
| CIMP.L | TCGA-AA-A01V | 0.634487 | 0.83404 | 0.090697 |
| CIMP.L | TCGA-AA-A01Z | 0.860295 | 0.860826 | 0.04449 |
| CIMP.L | TCGA-AA-A02Y | 0.729055 | 0.844909 | 0.046702 |
| CIMP.L | TCGA-AA-A03J | 0.819876 | 0.708634 | 0.431104 |
| CIMP.L | TCGA-AF-2689 | 0.61264 | 0.856744 | 0.213225 |
| CIMP.L | TCGA-AF-2691 | 0.876295 | 0.834919 | 0.400902 |
| CIMP.L | TCGA-AG-3584 | 0.917738 | 0.604282 | 0.742655 |
| CIMP.L | TCGA-AG-3598 | 0.177268 | 0.810807 | 0.144082 |
| CIMP.L | TCGA-AG-3599 | 0.912978 | 0.845274 | 0.055636 |
| CIMP.L | TCGA-AG-3605 | 0.283391 | 0.75301 | 0.036475 |
| CIMP.L | TCGA-AG-3608 | 0.917439 | 0.757617 | 0.053282 |
| CIMP.L | TCGA-AG-3878 | 0.810073 | 0.708474 | 0.538957 |
| CIMP.L | TCGA-AG-3881 | 0.60067 | 0.736803 | 0.032094 |
| CIMP.L | TCGA-AG-3885 | 0.42305 | 0.87093 | 0.02078 |
| CIMP.L | TCGA-AG-3902 | 0.495653 | 0.720849 | 0.141605 |
| CIMP.L | TCGA-AG-4007 | 0.920028 | 0.83698 | 0.804383 |
| CIMP.L | TCGA-AG-A011 | 0.767617 | 0.686586 | 0.526586 |
| non-CIMP | TCGA-A6-2670 | 0.9383 | 0.822606 | 0.723589 |
| non-CIMP | TCGA-A6-2677 | 0.943215 | 0.93207 | 0.581486 |
| non-CIMP | TCGA-A6-2678 | 0.83262 | 0.855306 | 0.765989 |
| non-CIMP | TCGA-A6-3808 | 0.841911 | 0.702792 | 0.39358 |
| non-CIMP | TCGA-AA-3514 | 0.882628 | 0.847183 | 0.677772 |
| non-CIMP | TCGA-AA-3519 | 0.881177 | 0.840652 | 0.770852 |
| non-CIMP | TCGA-AA-3520 | 0.287125 | 0.812662 | 0.039382 |
| non-CIMP | TCGA-AA-3521 | 0.469105 | 0.814705 | 0.02525 |
| non-CIMP | TCGA-AA-3526 | 0.859089 | 0.790744 | 0.667625 |
| non-CIMP | TCGA-AA-3534 | 0.898776 | 0.878602 | 0.016103 |
| non-CIMP | TCGA-AA-3544 | 0.889233 | 0.799572 | 0.690084 |
| non-CIMP | TCGA-AA-3548 | 0.529637 | 0.80689 | 0.393178 |
| non-CIMP | TCGA-AA-3552 | 0.398966 | 0.790401 | 0.056173 |
| non-CIMP | TCGA-AA-3560 | 0.741319 | 0.866348 | 0.022 |
| non-CIMP | TCGA-AA-3561 | 0.1541 | 0.922806 | 0.032269 |
| non-CIMP | TCGA-AA-3562 | 0.88764 | 0.821681 | 0.700065 |
| non-CIMP | TCGA-AA-3814 | 0.659126 | 0.735866 | 0.483481 |
| non-CIMP | TCGA-AA-3819 | 0.813792 | 0.704088 | 0.642723 |
| non-CIMP | TCGA-AA-3844 | 0.91843 | 0.866825 | 0.567977 |
| non-CIMP | TCGA-AA-3846 | 0.835608 | 0.77775 | 0.640919 |
| non-CIMP | TCGA-AA-3851 | 0.854258 | 0.763206 | 0.095994 |
| non-CIMP | TCGA-AA-3858 | 0.856956 | 0.805156 | 0.690482 |
| non-CIMP | TCGA-AA-3864 | 0.860785 | 0.791057 | 0.65417 |
| non-CIMP | TCGA-AA-3866 | 0.850966 | 0.748204 | 0.651978 |
| non-CIMP | TCGA-AA-3875 | 0.699085 | 0.683123 | 0.546302 |
| non-CIMP | TCGA-AA-3939 | 0.90322 | 0.804493 | 0.459296 |
| non-CIMP | TCGA-AA-3956 | 0.74782 | 0.685521 | 0.554196 |
| non-CIMP | TCGA-AA-3968 | 0.876266 | 0.809466 | 0.748273 |
| non-CIMP | TCGA-AA-3970 | 0.858609 | 0.659384 | 0.686369 |
| non-CIMP | TCGA-AA-3976 | 0.902283 | 0.847534 | 0.77263 |
| non-CIMP | TCGA-AA-3977 | 0.826478 | 0.743881 | 0.627202 |
| non-CIMP | TCGA-AA-3980 | 0.904541 | 0.777834 | 0.692148 |
| non-CIMP | TCGA-AA-3982 | 0.657565 | 0.670724 | 0.578813 |
| non-CIMP | TCGA-AA-3986 | 0.8469 | 0.691966 | 0.576784 |
| non-CIMP | TCGA-AA-A00F | 0.774385 | 0.711115 | 0.264544 |
| non-CIMP | TCGA-AA-A00K | 0.891189 | 0.74457 | 0.382151 |
| non-CIMP | TCGA-AA-A00L | 0.864589 | 0.891113 | 0.796013 |
| non-CIMP | TCGA-AA-A00W | 0.881259 | 0.804576 | 0.420816 |
| non-CIMP | TCGA-AA-A01F | 0.612167 | 0.91807 | 0.010281 |
| non-CIMP | TCGA-AA-A01K | 0.808829 | 0.673605 | 0.517253 |
| non-CIMP | TCGA-AA-A01T | 0.867319 | 0.819753 | 0.763594 |
| non-CIMP | TCGA-AA-A01X | 0.855069 | 0.808711 | 0.697709 |
| non-CIMP | TCGA-AA-A024 | 0.878918 | 0.837881 | 0.335726 |
| non-CIMP | TCGA-AA-A02E | 0.837441 | 0.878914 | 0.16227 |
| non-CIMP | TCGA-AA-A02H | 0.84144 | 0.818244 | 0.689766 |
| non-CIMP | TCGA-AA-A02K | 0.839407 | 0.743608 | 0.652416 |
| non-CIMP | TCGA-AG-3574 | 0.9304 | 0.882619 | 0.780109 |
| non-CIMP | TCGA-AG-3578 | 0.94643 | 0.863481 | 0.027987 |
| non-CIMP | TCGA-AG-3580 | 0.943984 | 0.770821 | 0.263681 |
| non-CIMP | TCGA-AG-3581 | 0.883494 | 0.801307 | 0.0246 |
| non-CIMP | TCGA-AG-3586 | 0.861949 | 0.818566 | 0.10745 |
| non-CIMP | TCGA-AG-3593 | 0.942628 | 0.890665 | 0.651873 |
| non-CIMP | TCGA-AG-3594 | 0.352756 | 0.758688 | 0.036975 |
| non-CIMP | TCGA-AG-3602 | 0.668692 | 0.752576 | 0.440755 |
| non-CIMP | TCGA-AG-3611 | 0.664033 | 0.907826 | 0.027068 |
| non-CIMP | TCGA-AG-3726 | 0.583427 | 0.512669 | 0.395147 |
| non-CIMP | TCGA-AG-3727 | 0.86182 | 0.825982 | 0.560795 |
| non-CIMP | TCGA-AG-3883 | 0.799559 | 0.737217 | 0.587384 |
| non-CIMP | TCGA-AG-3890 | 0.846974 | 0.766601 | 0.17468 |
| non-CIMP | TCGA-AG-3894 | 0.884457 | 0.819733 | 0.540069 |
| non-CIMP | TCGA-AG-3896 | 0.802404 | 0.790912 | 0.419717 |
| non-CIMP | TCGA-AG-3898 | 0.778021 | 0.733107 | 0.349146 |
| non-CIMP | TCGA-AG-3901 | 0.785377 | 0.553092 | 0.37813 |
| non-CIMP | TCGA-AG-3909 | 0.758582 | 0.801792 | 0.712319 |
| non-CIMP | TCGA-AG-3999 | 0.225052 | 0.846021 | 0.075346 |
| non-CIMP | TCGA-AG-4005 | 0.534181 | 0.835085 | 0.028822 |
| non-CIMP | TCGA-AG-4008 | 0.848971 | 0.791535 | 0.081462 |
| non-CIMP | TCGA-AG-A002 | 0.145796 | 0.852045 | 0.018252 |
| non-CIMP | TCGA-AG-A00C | 0.876185 | 0.819587 | 0.632309 |
| non-CIMP | TCGA-AG-A00Y | 0.692546 | 0.828593 | 0.398447 |
| non-CIMP | TCGA-AG-A015 | 0.222283 | 0.859382 | 0.01335 |
| non-CIMP | TCGA-AG-A016 | 0.779629 | 0.848347 | 0.830837 |
| non-CIMP | TCGA-AG-A01L | 0.894403 | 0.701314 | 0.486766 |
| non-CIMP | TCGA-AG-A025 | 0.818167 | 0.760383 | 0.028649 |
| non-CIMP | TCGA-AG-A026 | 0.859157 | 0.781934 | 0.618612 |
| non-CIMP | TCGA-AY-4070 | 0.695944 | 0.768176 | 0.081382 |
| non-CIMP | TCGA-AY-4071 | 0.924587 | 0.840596 | 0.335078 |
| non-CIMP | TCGA-A6-2674 | 0.771669 | 0.602821 | 0.362814 |
| non-CIMP | TCGA-A6-3807 | 0.850338 | 0.737026 | 0.668266 |
| non-CIMP | TCGA-A6-3810 | 0.894819 | 0.818813 | 0.727079 |
| non-CIMP | TCGA-AA-3517 | 0.905159 | 0.873396 | 0.78842 |
| non-CIMP | TCGA-AA-3524 | 0.895719 | 0.853276 | 0.768749 |
| non-CIMP | TCGA-AA-3529 | 0.831226 | 0.784053 | 0.672593 |
| non-CIMP | TCGA-AA-3530 | 0.699024 | 0.854501 | 0.270856 |
| non-CIMP | TCGA-AA-3531 | 0.925658 | 0.921256 | 0.835227 |
| non-CIMP | TCGA-AA-3532 | 0.865454 | 0.675753 | 0.489086 |
| non-CIMP | TCGA-AA-3538 | 0.493836 | 0.785384 | 0.034726 |
| non-CIMP | TCGA-AA-3542 | 0.66548 | 0.916553 | 0.474676 |
| non-CIMP | TCGA-AA-3553 | 0.859807 | 0.620915 | 0.187418 |
| non-CIMP | TCGA-AA-3554 | 0.514746 | 0.636218 | 0.055493 |
| non-CIMP | TCGA-AA-3666 | 0.14749 | 0.844846 | 0.037984 |
| non-CIMP | TCGA-AA-3667 | 0.886781 | 0.82893 | 0.728244 |
| non-CIMP | TCGA-AA-3678 | 0.762301 | 0.68087 | 0.068265 |
| non-CIMP | TCGA-AA-3679 | 0.848204 | 0.801721 | 0.445692 |
| non-CIMP | TCGA-AA-3685 | 0.842728 | 0.732777 | 0.640488 |
| non-CIMP | TCGA-AA-3688 | 0.899575 | 0.871668 | 0.768062 |
| non-CIMP | TCGA-AA-3692 | 0.837405 | 0.804368 | 0.720546 |
| non-CIMP | TCGA-AA-3693 | 0.914732 | 0.896381 | 0.795682 |
| non-CIMP | TCGA-AA-3696 | 0.865698 | 0.827039 | 0.729408 |
| non-CIMP | TCGA-AA-3811 | 0.602192 | 0.824805 | 0.294221 |
| non-CIMP | TCGA-AA-3812 | 0.616446 | 0.553934 | 0.476456 |
| non-CIMP | TCGA-AA-3856 | 0.469526 | 0.641391 | 0.043362 |
| non-CIMP | TCGA-AA-3860 | 0.828717 | 0.785256 | 0.662172 |
| non-CIMP | TCGA-AA-3862 | 0.88073 | 0.773812 | 0.079299 |
| non-CIMP | TCGA-AA-3867 | 0.292663 | 0.544451 | 0.023 |
| non-CIMP | TCGA-AA-3869 | 0.465395 | 0.815711 | 0.038138 |
| non-CIMP | TCGA-AA-3872 | 0.272562 | 0.719411 | 0.042574 |
| non-CIMP | TCGA-AA-3952 | 0.871223 | 0.79392 | 0.645215 |
| non-CIMP | TCGA-AA-3955 | 0.908584 | 0.850689 | 0.335012 |
| non-CIMP | TCGA-AA-3971 | 0.785846 | 0.715121 | 0.6019 |
| non-CIMP | TCGA-AA-3973 | 0.916613 | 0.890628 | 0.835226 |
| non-CIMP | TCGA-AA-3975 | 0.860374 | 0.773367 | 0.746999 |
| non-CIMP | TCGA-AA-3984 | 0.866238 | 0.680924 | 0.57255 |
| non-CIMP | TCGA-AA-A00N | 0.74022 | 0.67963 | 0.078932 |
| non-CIMP | TCGA-AA-A00O | 0.658347 | 0.702454 | 0.520896 |
| non-CIMP | TCGA-AA-A00Z | 0.783705 | 0.74445 | 0.623494 |
| non-CIMP | TCGA-AA-A010 | 0.777438 | 0.781026 | 0.401226 |
| non-CIMP | TCGA-AA-A017 | 0.838575 | 0.715205 | 0.583263 |
| non-CIMP | TCGA-AA-A01C | 0.855417 | 0.788894 | 0.647264 |
| non-CIMP | TCGA-AA-A01S | 0.901667 | 0.8829 | 0.819002 |
| non-CIMP | TCGA-AA-A02F | 0.909505 | 0.861803 | 0.698148 |
| non-CIMP | TCGA-AA-A02J | 0.879571 | 0.79727 | 0.698616 |
| non-CIMP | TCGA-AA-A02W | 0.158247 | 0.795085 | 0.034217 |
| non-CIMP | TCGA-AF-2692 | 0.887304 | 0.827043 | 0.077799 |
| non-CIMP | TCGA-AF-3400 | 0.640448 | 0.581668 | 0.041139 |
| non-CIMP | TCGA-AF-3913 | 0.808769 | 0.851349 | 0.162615 |
| non-CIMP | TCGA-AG-3582 | 0.949938 | 0.886047 | 0.307935 |
| non-CIMP | TCGA-AG-3583 | 0.886619 | 0.799175 | 0.663868 |
| non-CIMP | TCGA-AG-3587 | 0.307902 | 0.915832 | 0.397355 |
| non-CIMP | TCGA-AG-3601 | 0.71325 | 0.906843 | 0.720416 |
| non-CIMP | TCGA-AG-3609 | 0.905228 | 0.828731 | 0.714079 |
| non-CIMP | TCGA-AG-3612 | 0.869869 | 0.863729 | 0.056697 |
| non-CIMP | TCGA-AG-3728 | 0.868917 | 0.797804 | 0.525096 |
| non-CIMP | TCGA-AG-3882 | 0.867719 | 0.740816 | 0.628863 |
| non-CIMP | TCGA-AG-3887 | 0.858682 | 0.840013 | 0.752688 |
| non-CIMP | TCGA-AG-3892 | 0.770523 | 0.686445 | 0.492581 |
| non-CIMP | TCGA-AG-3893 | 0.924616 | 0.835874 | 0.525548 |
| non-CIMP | TCGA-AG-4001 | 0.679144 | 0.535475 | 0.372569 |
| non-CIMP | TCGA-AG-4015 | 0.660401 | 0.874476 | 0.035453 |
| non-CIMP | TCGA-AG-A008 | 0.907922 | 0.897503 | 0.318326 |
| non-CIMP | TCGA-AG-A00H | 0.621031 | 0.682444 | 0.254664 |
| non-CIMP | TCGA-AG-A014 | 0.965628 | 0.86333 | 0.424394 |
| non-CIMP | TCGA-AG-A01J | 0.775982 | 0.682599 | 0.369804 |
| non-CIMP | TCGA-AG-A01N | 0.823483 | 0.43506 | 0.032458 |
| non-CIMP | TCGA-AG-A023 | 0.740112 | 0.661134 | 0.061483 |
| non-CIMP | TCGA-AG-A02G | 0.916532 | 0.846558 | 0.364527 |
| non-CIMP | TCGA-AG-A032 | 0.861213 | 0.734318 | 0.030811 |
